# Ethnic variations in metabolic syndrome components and their associations with the gut microbiota: the HELIUS study

**DOI:** 10.1186/s13073-024-01295-7

**Published:** 2024-03-20

**Authors:** Manon Balvers, Marcus de Goffau, Natal van Riel, Bert-Jan van den Born, Henrike Galenkamp, Koos Zwinderman, Max Nieuwdorp, Evgeni Levin

**Affiliations:** 1 Department of Internal and Vascular Medicine, Amsterdam University Medical Centers, Amsterdam, The Netherlands; 2 Tytgat Institute for Liver and Intestinal Research, Amsterdam University Medical Centers, Amsterdam, The Netherlands; 3 HORAIZON Technology BV, Marshallaan 2, Delft, 2625 GZ The Netherlands; 4 Department of Public and Occupational Health and Amsterdam Public Health Research Institute, Amsterdam University Medical Centers, Amsterdam, The Netherlands; 5 Department of Clinical Epidemiology and Biostatistics, Amsterdam University Medical Centers, Amsterdam, 1105 AZ The Netherlands

**Keywords:** Metabolic syndrome, Ethnicity, Gut microbiome, Christensenellaceae, α-diversity, HELIUS study

## Abstract

**Background:**

The occurrence of metabolic syndrome (MetS) and the gut microbiota composition are known to differ across ethnicities yet how these three factors are interwoven is unknown. Also, it is unknown what the relative contribution of the gut microbiota composition is to each MetS component and whether this differs between ethnicities. We therefore determined the occurrence of MetS and its components in the multi-ethnic HELIUS cohort and tested the overall and ethnic-specific associations with the gut microbiota composition.

**Methods:**

We included 16,209 treatment naïve participants of the HELIUS study, which were of Dutch, African Surinamese, South-Asian Surinamese, Ghanaian, Turkish, and Moroccan descent to analyze MetS and its components across ethnicities. In a subset (*n* = 3443), the gut microbiota composition (16S) was associated with MetS outcomes using linear and logistic regression models.

**Results:**

A differential, often sex-dependent, prevalence of MetS components and their combinations were observed across ethnicities. Increased blood pressure was commonly seen especially in Ghanaians, while South-Asian Surinamese and Turkish had higher MetS rates in general and were characterized by worse lipid-related measures. Regarding the gut microbiota, when ethnic-independent associations were assumed, a higher α-diversity, higher abundance of several ASVs (mostly for waist and triglyceride-related outcomes) and a trophic network of ASVs of *Ruminococcaceae*, *Christensenellaceae*, and *Methanobrevibacter* (RCM) bacteria were associated with better MetS outcomes. Statistically significant ethnic-specific associations were however noticed for α-diversity and the RCM trophic network. Associations were significant in the Dutch but not always in all other ethnicities. In Ghanaians, a higher α-diversity and RCM network abundance showed an aberrant positive association with high blood pressure measures compared to the other ethnicities. Even though adjustment for socioeconomic status-, lifestyle-, and diet-related variables often attenuated the effect size and/or the statistical significance of the ethnic-specific associations, an overall similar pattern across outcomes and ethnicities remained.

**Conclusions:**

The occurrence of MetS characteristics among ethnicities is heterogeneous. Both ethnic-independent and ethnic-specific associations were identified between the gut microbiota and MetS outcomes. Across multiple ethnicities, a one-size-fits-all approach may thus be reconsidered in regard to both the definition and/or treatment of MetS and its relation to the gut microbiota.

**Supplementary Information:**

The online version contains supplementary material available at 10.1186/s13073-024-01295-7.

## Background

Metabolic syndrome (MetS) is a risk factor for type 2 diabetes (T2D) and cardiovascular disease (CVD), which are increasingly among the main causes of morbidity and mortality worldwide. MetS represents the clustering of individual risk factors, including hypertension, central obesity, dysglycemia, and dislipidaemia [1, 2]. The exact pathogenic mechanism is not exactly known, yet insulin resistance is proposed as the underlying factor [2]. Which exact diagnostic criteria should be used is still under debate, as is the question whether MetS can be considered a single syndrome or represents multiple syndromes with different cardiovascular risk profiles [2–4].

Differences across ethnicities exist in the prevalence of MetS itself as well as in the prevalence of the individual components that are included in the MetS definition. For example, African American people have a higher prevalence of hypertension [5], while they suffer less often from dyslipidaemia [6] compared to their Caucasian counterparts. Lower cut-offs for central obesity are already used for males from South-Asian descent [2]. Furthermore, triglyceride levels were not considered to be associated with insulin resistance in African Americans, and Gurka et al. (2014) mentioned different correlations for the individual components with the underlying MetS construct across ethnicities [7, 8]. Next to genetic or biological aspects, (self-reported) ethnicity also entails societal, behavioral, and environmental factors [9–11]. As the prevalence of MetS is often influenced by such factors, including socioeconomic status, diet, physical activity, and educational level [1], this often complicates the interpretation of health disparities across ethnic groups.

Another environmental factor that is linked to MetS and which exhibits a different composition across ethnicities is the gut microbiome [12]. The gut microbiome, composed of trillions of bacteria, fungi, viruses, and their corresponding genes, has previously been proposed to be associated with insulin resistance [13]. Several studies have already identified associations between the gut microbiome and MetS and/or its components, which are proposed to be established mainly via inflammation and metabolism modulation [14, 15]. In addition, a fecal microbiota transplantation (FMT) derived from lean donors given to obese Dutch males with MetS showed a temporarily improvement in insulin sensitivity after 6 weeks compared to males receiving their own fecal microbiota, highlighting the potential therapeutic effect of the gut microbiota in MetS [13].

To gain more insight in the effect of ethnicity, including rarely studied ethnic minorities, on the occurrence of MetS, its individual components, and the combination of these risk factors, we used the Healthy Life in Urban Setting (HELIUS) cohort [16, 17] in Amsterdam, the Netherlands. Furthermore, we analyzed the link between the gut microbiota and MetS and its components in a subgroup of this cohort of which gut microbial sequencing data was available. Those insights could help to evaluate if a one-size-fits-all approach for MetS is still appropriate in regard to its definition, treatment, and the role of the gut microbiota across different ethnicities.

## Methods

### Study population

The HELIUS study is an ongoing prospective cohort study in Amsterdam, the Netherlands, which at baseline included 18–70 years old residents. Participants were randomly recruited from the municipal registry, after being stratified by their ethnic origin, being of either Surinamese, Ghanaian, Turkish, Moroccan, or Dutch descent. A detailed description of the study population, study design, and rationale are provided elsewhere [16, 17]. The Academic Medical Center (AMC) Medical Ethics Committee approved the HELIUS study, and all participants provided written informed consent.

Of the total 24,789 baseline participants, a number of 22,165 people participated in the physical examination, including collection of biological samples, and filled in the questionnaire as described in Snijder et al. [16]. Out of these 22,165 participants, we excluded Javanese Surinamese (*n* = 233), other Surinamese (*n* = 267), and those of other/unknown ethnic origin (*n* = 48) due to insufficient numbers of these ethnicities. We further excluded participants with missing data on the components of MetS or participants with diabetes (defined by either the use of antidiabetic medication, fasting HbA1c levels ≥ 48 mmol/L or fasting glucose levels ≥ 7.0 mmol/L, or with missing values for those criteria), and all participants on either antihypertensive or antilipidemic medication or unknown medication usage, leaving 16,209 participants for the total dataset.

For the analysis on the gut microbiota composition, we included the subset of the participants from the total dataset in whom gut microbiota data were available after quality control of this data (see below) [18]. Participants who used antibiotics in the past 3 months or of unknown use were excluded. A number of 3443 participants were finally included in the gut microbiota dataset.

### Baseline data collection

After a positive response, subjects received a confirmation letter of an appointment for a physical examination and a digital or paper version of the questionnaire (depending on the preference of the subject) to fill out at home. At the research locations, participants underwent a physical examination, during which measurements of blood pressure and anthropometric (e.g., weight, height and waist circumference) characteristics were obtained. Measures of waist circumference, systolic blood pressure, and diastolic blood pressure were performed in duplicate and then averaged. Furthermore, participants were asked to bring their prescribed medications, which were coded according to the Anatomical Therapeutic Chemical (ATC) classification. Fasting blood samples were drawn after an overnight fast and were analyzed by the main laboratory department of the Academic Medical Center in Amsterdam to determine glucose, lipid (total cholesterol, HDL-cholesterol and triglyceride levels), and HbA1c profiles. More detailed information about the measurements is described elsewhere [19].

### Ethnicity

Ethnicity of the participant was defined according to his/her country of birth as well as that of his/her parents, which is currently the most widely accepted and most valid assessment of ethnicity in the Netherlands [20]. Specifically, a participant is considered to be of non-Dutch ethnic origin if he/she fulfills either of the following criteria: (1) he or she was born in another country and has at least one parent born in another country (first generation) or (2) he or she was born in the Netherlands but both his/her parents were born in another country (second generation). Of the Surinamese immigrants in the Netherlands, approximately 80% are of either African or South-Asian origin. After data collection, Surinamese subgroups were classified according to self-reported ethnic origin. Participants were considered to be of Dutch origin if the person and both parents were born in the Netherlands.

### Gut microbiota profiling and processing

Stool samples were collected, sequenced, and processed as previously described in detail in another study [21]. In short, DNA was extracted from the home-collected stool samples (*n* = 6056) after which the V4 region of the 16S rRNA gene was sequenced on an Illumina MiSeq instrument. After merging paired-end reads and quality filtering the raw reads with USEARCH [22] (v11.0.667_i86linux64), an Amplicon Sequence Variant (ASV) table was obtained using the UNOISE3 algorithm from USEARCH. Taxonomy was assigned with “dada2” [23] (v1.12.1) on the SILVA reference database [24] (v.132), and a phylogenetic tree was obtained using MAFFT [25, 26] (v. 7.427) and FastTree [27] (v. 2.1.11). In the end, the ASV table was rarefied to 14,932 counts per sample. Out of the 6056 sequenced samples, 6032 samples remained after the total quality control and were used as starting point for the above-described inclusion in our gut microbiota cohort.

### MetS definition

MetS definition was based on the definition by Alberti et al. [2]. Participants were classified as having MetS, if they fulfilled at least 3 of the following criteria:

1. High blood pressure, defined by systolic blood pressure ≥ 130 mmHg and/or diastolic blood pressure ≥ 85 mmHg
2. Central obesity, defined by waist circumference ≥ 80 cm (in females) or ≥ 90 cm (in males from South-Asian Surinamese descent) or ≥ 94 cm (in males not from South-Asian Surinamese descent)
3. High triglycerides, defined by triglycerides ≥ 1.7 mmol/L
4. High glucose, defined by glucose ≥ 5.6 mmol/L
5. Low HDL, defined by HDL cholesterol < 1.29 mmol/L (in females) or < 1.03 mmol/L (in males)

The same criteria were used during the analysis on the individual components of MetS.

### Covariates

Apart from age and sex, we considered the following covariates obtained via the questionnaire: socioeconomic status (highest obtained educational level, occupational level and employment status), lifestyle (physical activity, smoking and alcohol use), and dietary habits (sugar intake and fruit intake). In gut microbiota analyses, we also took proton pump inhibitor (PPI) use into account, as this is a known confounder of the gut microbiota.

The highest educational level obtained in the Netherlands or in the country of origin was categorized as higher (higher vocational schooling or university), intermediate (intermediate vocational schooling or intermediate/higher secondary schooling), lower (lower vocational schooling or lower secondary schooling), or elementary (never been to school or elementary schooling only). Current employment status was indicated as either working, not in work force, unemployed, or unable to work. The categories academic, higher, intermediate, lower, and elementary were used to indicate occupational status. For the lifestyle-related variables, we used a binary indicator for physical activity (i.e., 30 min of moderate/intensive exercise for at least 5 days a week, which is conform the Dutch Standard for Health exercise) and alcohol use (used alcohol in the last 12 months). Smoking was categorized into yes, former, and never. Since we did not have the same Food Frequency Questionnaire for all ethnicities, we derived composite variables as proxies for dietary habits. We used regularly fruit intake (yes/no) as a proxy for a healthy diet, which was indicated as eating at least one piece of fruit for at least 5 days/week. In regard to an unhealthy diet, we used the daily ingestion (yes/no) of sugar drinks as a proxy. This variable was considered to be present if participants responded that they had a daily consumption of either fruit juice, tea with sugar, regular soft drink, sports drink, fruit syrup, fruit drink, malt beer, or coffee with sugar or when a participant consumed 7 of those drinks 1 to 6 days a week.

### Statistical analysis

Clinical and anthropometric values are summarized as mean ± standard deviation or as median (interquartile range) for normally and non-normally distributed values, respectively. Categorical variables are presented with either counts or percentages.

For the subsequent analyses, except for analyses on combinations of components, all analyses were performed for the binarized outcomes of all MetS components and MetS itself as well as on the continuous outcomes of the components.

Differences in MetS outcomes across ethnicities were assessed with general linear models (GLM) (family “binomial” for binarized outcomes, family “gaussian” for continuous outcomes). Models were run for the total dataset and adjusted for age and sex (male as reference). Statistical significance of the ethnicity variable (Dutch as reference) was assessed with the likelihood ratio test (LRT). In addition, potential sex-dependent ethnic differences in MetS outcomes were tested with the inclusion of an interaction term between sex and ethnicity in the previous model, again using a LRT. To assess the potential influence of known confounders on the MetS outcomes, the same models were subsequently run with adjustment for socioeconomic factors, lifestyle, and dietary habits, in which higher educational level, academic occupational level, working employment status, never smoked, no alcohol use, no regular physical activity, no regular fruit intake, and no daily sugar drinks intake were set as reference.

Differences in prevalence of all possible combinations of components across ethnicities were assessed with the chi-squared test, performed separately on males and females from both the total dataset and MetS only subjects.

Analyses on the gut microbiota composition were only performed on samples from the gut microbiota dataset. The diversity of the gut microbiota per participant was indicated with several α-diversity indices calculated at the ASV level, including Shannon index (R package vegan 2.6–4 [28]; function “diversity”), richness (number of unique ASVs; R package vegan; function “specnumber”), and Faith’s PD (R package picante v.1.8.2 [29]; function “pd”). To assess the effect of α-diversity on MetS outcomes, logistic regression (GLM with binomial family; for binarized outcomes) and linear regression (GLM with gaussian family; for continuous outcome) were performed for each diversity index separate (independent variable). Triglyceride levels were log transformed to account for their non-normal distribution. Models were adjusted for age, sex, ethnicity, and the interaction between sex and ethnicity (if this interaction was significant during analyses on the total cohort), assuming an ethnic-independent effect of α-diversity (i.e., ethnic-independent model). To test if the effect of α-diversity on the outcomes was different across ethnicities, an interaction term between ethnicity and α-diversity was added to the ethnic-independent model and tested for significance with a LRT. Those models were considered as baseline models (model 1). In addition, additive adjustment for socioeconomic factors (model 2; model 1 + socioeconomic), lifestyle-related variables (model 3; i.e., model 2 + lifestyle), and dietary-related variables (model 4; i.e., model 3 + diet) was performed to assess the influence of known confounders on the MetS outcomes. We also adjusted for PPI use in models 2, 3, and 4, since this is a known confounder of the gut microbiome composition. Coefficients and standard errors for each ethnicity were obtained from the model output, including the coefficients and variance–covariance matrix, if the interaction was significant.

Similar to the α-diversity, we also assessed the effect of individual ASVs in regard to MetS outcomes. To account for the bias in ethnic sample size, ASVs were included if they fulfilled the following criteria in at least one ethnicity, in either males or females: present in > 5% of the samples and a mean relative abundance > 0.02%. This resulted in the inclusion of 604 ASVs. ASVs were included in the models as arcsin square-root transformed relative abundance, to account for the non-normality of the distribution. The same ethnic-independent models (i.e., logistic or linear regression, adjusted for age, sex, ethnicity, and optionally sex:ethnicity as baseline models, and additional adjusted for PPI use, socioeconomic, lifestyle, and diet variables) were performed for all ASVs (independent variable). Per outcome, either binarized or continuous, correction for multiple testing was performed using the Benjamini–Hochberg correction (p.adjust) [30]. All ASVs were also tested for ethnic specific effects by including an interaction term between ethnicity and ASV to the ethnic-independent models and tested for significance with a LRT. Correction for multiple comparisons was performed in a similar manner as described above.

Subsequently, an analysis was performed on the ASVs that were significant for at least 3 components (combining binary and continuous outcomes and considering MetS itself as a component) in the ethnic-independent models. ASVs were clustered based on their Spearman’s correlation, using hierarchical linkage clustering (Euclidian distance, average agglomeration method) with hclust. Abundances of ASVs belonging to clusters were summed, arcsin square-root transformed, and tested for effects on MetS outcomes in the same way as the α-diversity measures.

Statistical analyses were performed in R 4.0.3 [31] (using RStudio v 1.3.1093). *p*-values < 0.05 (either BH adjusted (ASVs) or unadjusted (other models); either for single terms or interaction terms) were considered to be statistically significant.

## Results

In total, we included 16,209 treatment naïve subjects across six ethnicities for whom the characteristics are displayed in Table 1.

**Table 1:** Population characteristics for the total population cohort. Overview of population characteristics for the Dutch, South-Asian Surinamese (SA Surinamese), African Surinamese (Afr Surinamese), Ghanaian, Turkish, and Moroccan, presented separately per sex

	Dutch	SA Surinamese	Afr Surinamese	Ghanaian	Turkish	Moroccan
**Males**						
*N*	1717	863	1145	570	1301	1257
Age (in years)	44.45 ± 13.35	39.07 ± 12.48	44.91 ± 12.87	43.72 ± 12.05	38.26 ± 11.47	39.68 ± 11.92
Educational level						
Higher (%)	63.9	27.1	20.3	9.5	17.9	20.5
Intermediate (%)	23.0	36.5	34.8	27.9	30.7	35.2
Lower (%)	10.4	26.9	38.5	44.4	30.7	22.3
Elementary (%)	2.3	9.2	5.7	16.3	19.8	20.4
NA (%)	0.4	0.3	0.7	1.9	0.9	1.5
Occupational level						
Academic (%)	22.9	6.6	2.5	1.2	3.8	2.8
Higher (%)	37.8	18.3	17.0	3.5	9.9	12.7
Intermediate (%)	21.0	26.5	23.4	8.8	21.1	22.2
Lower (%)	11.4	32.1	38.8	26.5	41.7	38.7
Elementary (%)	0.9	5.9	8.6	45.4	10.4	12.8
NA (%)	6.1	10.5	9.7	14.6	13.1	10.8
Employment status						
Working (%)	79.6	70.2	67.8	72.3	70.4	69.9
Not in workforce (%)	12.1	9.4	7.9	6.8	7.3	7.0
Unemployed (%)	5.8	13.8	16.9	15.6	14.7	15.4
Unfit for work (%)	2.3	5.1	6.0	3.3	5.9	7.2
NA (%)	0.2	1.5	1.3	1.9	1.7	0.6
Smoking						
Yes (%)	26.0	40.0	45.3	8.1	42.1	27.3
Never (%)	36.2	45.1	37.3	79.5	34.7	48.8
Former (%)	37.8	14.6	16.9	11.8	22.5	23.4
NA (%)	0.1	0.3	0.4	0.7	0.7	0.5
Alcohol						
Yes (%)	95.0	69.9	80.1	53.9	36.7	14.2
No (%)	5.0	29.8	19.1	45.1	62.4	85.2
NA (%)	0	0.3	0.8	1.1	0.9	0.6
Physical activity						
Yes (%)	72.5	55.7	69.1	62.1	49.7	54.4
No (%)	27.4	43.9	30.8	37.9	50.0	45.3
NA (%)	0.1	0.3	0.1	0	0.4	0.2
SugarDrinks						
Yes (%)	49.7	67.4	70.8	49.5	69.5	74.3
No (%)	50.1	32.1	28.1	48.1	29.0	25.1
NA (%)	0.2	0.5	1.0	2.5	1.5	0.6
Fruit intake						
Yes (%)	56.6	39.9	40.2	34.0	43.0	39.6
No (%)	43.3	60.0	59.3	64.9	56.3	59.4
NA (%)	0.1	0.1	0.5	1.1	0.7	0.7
PPI use						
Yes (%)	3.4	3.9	3.5	2.5	7.5	6.6
MetSyn = yes (%)	20.6	29.2	15.4	13.3	32.4	24.4
Central obesity = yes (%)	37.8	51.1	31.3	31.9	55.0	48.0
High glucose = yes (%)	29.7	34.1	24.3	22.6	28.1	31.8
High blood pressure = yes (%)	43.5	42.3	54.5	63.5	41.5	38.2
Low HDL = yes (%)	11.2	27.2	11.5	7.0	33.1	25.9
High triglycerides = yes (%)	14.5	19.6	7.2	4.4	25.1	14.1
Waist circumference (in cm)	91.90 ± 10.60	90.79 ± 11.76	89.68 ± 11.40	89.19 ± 10.16	95.54 ± 11.48	93.73 ± 10.94
Fasting glucose (in mmol/L)	5.34 ± 0.47	5.42 ± 0.47	5.25 ± 0.47	5.21 ± 0.48	5.35 ± 0.45	5.38 ± 0.48
SBP (in mmHg)	127.9 ± 14.7	126.6 ± 14.2	131.3 ± 16.5	135.4 ± 17.1	125.6 ± 12.8	125.6 ± 13.1
DBP (in mmHg)	80.3 ± 9.6	80.9 ± 10.0	82.9 ± 10.5	85.4 ± 11.4	80.3 ± 9.3	78.3 ± 8.8
HDL (in mmol/L)	1.41 ± 0.36	1.20 ± 0.32	1.40 ± 0.38	1.52 ± 0.43	1.15 ± 0.30	1.20 ± 0.30
Triglycerides (in mmol/L)	0.9 [0.63–1.32]	1.05 [0.72–1.47]	0.75 [0.52–1.1]	0.68 [0.49–0.96]	1.09 [0.73–1.71]	0.9 [0.63–1.33]
**Females**						
*N*	2113	1113	1615	925	1571	2019
Age (in years)	43.59 ± 13.79	41.31 ± 12.51	43.57 ± 12.16	40.23 ± 10.80	37.14 ± 11.14	36.97 ± 12.02
Educational level						
Higher (%)	64.8	26.8	28.7	5.9	16.3	18.6
Intermediate (%)	20.5	32.0	41.7	23.0	32.5	36.7
Lower (%)	11.7	30.6	25.8	37.0	19.7	16.4
Elementary (%)	2.4	10.0	3.0	32.0	30.4	27.7
NA (%)	0.5	0.6	0.9	2.1	1.1	0.6
Occupational level						
Academic (%)	19.4	5.3	2.8	0.6	3.3	2.4
Higher (%)	38.1	16.2	20.6	2.6	9.2	12.4
Intermediate (%)	20.9	31.4	39.4	8.4	19.4	23.2
Lower (%)	13.4	27.3	22.8	18.3	22.5	17.1
Elementary (%)	1.7	7.6	3.9	52.2	15.1	10.4
NA (%)	6.6	12.1	10.3	17.8	30.4	34.5
Employment status						
Working (%)	75.9	65.9	67.5	56.0	44.1	42.0
Not in workforce (%)	15.8	14.3	11.0	8.5	33.2	37.6
Unemployed (%)	5.4	12.6	14.1	25.0	14.1	14.1
Unfit for work (%)	2.6	6.5	6.7	7.4	7.1	5.2
NA (%)	0.3	0.8	0.8	3.1	1.5	1.1
Smoking						
Yes (%)	23.2	21.1	25.6	2.2	30.7	6.2
Never (%)	40.3	67.7	58.3	93.1	57.2	90.0
Former (%)	36.1	10.9	15.7	4.1	11.6	3.5
NA (%)	0.3	0.3	0.4	0.6	0.4	0.2
Alcohol						
Yes (%)	89.9	53.5	68.3	44.1	14.6	4.6
No (%)	9.9	46.2	31.5	54.6	84.9	95.1
NA (%)	0.2	0.3	0.2	1.3	0.5	0.3
Physical activity						
Yes (%)	78.2	46.3	54.4	46.3	35.2	39.7
No (%)	21.7	53.5	45.5	53.6	64.7	60.1
NA (%)	0.1	0.3	0.1	0.1	0.1	0.1
SugarDrinks						
Yes (%)	34.4	57.3	56.2	53.7	56.3	61.1
No (%)	65.1	41.1	42.3	42.7	42.0	37.5
NA (%)	0.5	1.6	1.5	3.6	1.7	1.3
Fruit intake						
Yes (%)	70.5	52.7	50.5	33.8	54.7	49.3
No (%)	29.3	46.5	48.9	65.0	44.6	49.8
NA (%)	0.2	0.8	0.6	1.2	0.7	0.9
PPI use						
Yes (%)	3.5	5.7	4.6	2.7	10.0	7.4
MetSyn = yes (%)	9.0	20.6	14.9	13.5	18.0	15.0
Central obesity = yes (%)	56.4	68.9	74.1	80.8	72.6	72.7
High glucose = yes (%)	11.4	17.7	11.8	11.5	11.2	14.1
High blood pressure = yes (%)	19.2	26.6	39.0	45.8	18.0	15.6
Low HDL = yes (%)	12.7	33.8	21.2	15.8	36.4	33.5
High triglycerides = yes (%)	5.2	8.5	2.0	1.3	9.9	4.5
Waist circumference (in cm)	83.77 ± 11.50	86.83 ± 12.97	89.68 ± 14.11	91.47 ± 13.05	89.29 ± 13.82	89.75 ± 14.00
Fasting glucose (in mmol/L)	5.02 ± 0.45	5.13 ± 0.49	5.00 ± 0.46	4.99 ± 0.47	5.03 ± 0.44	5.05 ± 0.47
SBP (in mmHg)	118.2 ± 14.7	120.4 ± 16.9	125.0 ± 16.9	129.4 ± 18.5	116.5 ± 13.8	115.7 ± 13.7
DBP (in mmHg)	73.2 ± 8.9	75.7 ± 9.9	78.3 ± 10.5	80.4 ± 10.9	73.4 ± 8.9	71.3 ± 8.4
HDL (in mmol/L)	1.76 ± 0.44	1.48 ± 0.38	1.63 ± 0.43	1.69 ± 0.43	1.45 ± 0.36	1.46 ± 0.34
Triglycerides (in mmol/L)	0.71 [0.5–0.99]	0.81 [0.6–1.14]	0.63 [0.46–0.87]	0.54 [0.41–0.73]	0.82 [0.56–1.19]	0.67 [0.46–0.99]

### Heterogeneous and sex-dependent patterns emerge across ethnicities for individual MetS outcomes

**Fig. 1:**
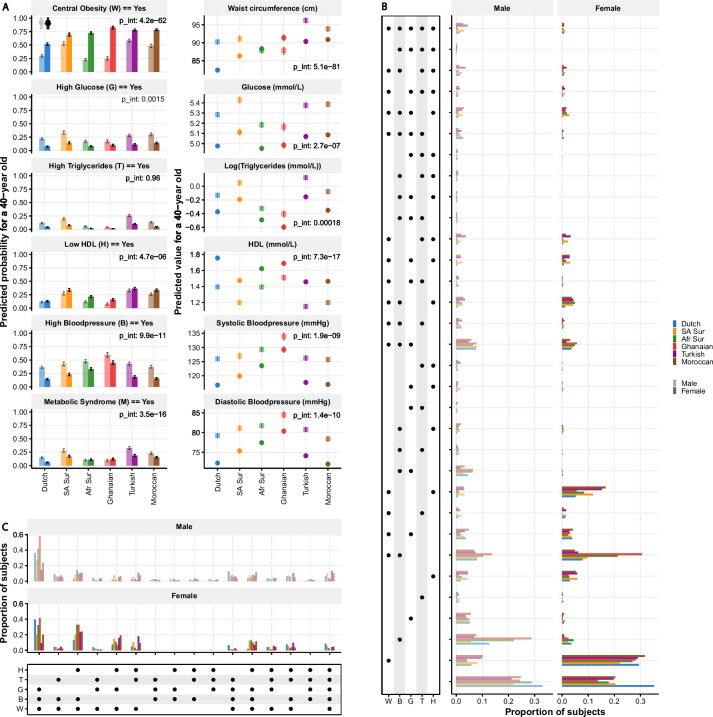
Overview of the occurrence of the metabolic syndrome (MetS)-related measures in the total population (*n* = 16,209). **A** Predicted outcomes (with 95% CI) for the (logistic) regression models with each outcome measure predicted on age, sex, ethnicity, and (except for HighTri) sex:ethnicity. Values are provided for a 40 years old person from the different groups. *p*-values for the interaction term (tested with a likelihood ratio test) in the model are stated. The left column represents the binarized outcomes; the right column represents the continuous outcomes. **B** Prevalence of each possible combination of individual (binarized) metabolic syndrome components for the total population indicated per sex and ethnicity, not adjusted for age. The components present in each specific combination are indicated by the black dots in the left part of the figure. The proportion of subjects with a particular combination within each group is indicated by the bars on the right part of the figure. W = central obesity, B = high blood pressure, H = low HDL, T = high triglycerides, G = high glucose. **C** Prevalence of each possible combination of individual (binarized) metabolic syndrome components for the MetS population indicated per sex and ethnicity, not adjusted for age. The components present in each specific combination are indicated by the black dots in the bottom part of the figure. The proportion of subjects with a particular combination is indicated by the bars at the top part of the figure. W = central obesity, B = high blood pressure, H = low HDL, T = high triglycerides, G = high glucose

Both ethnicity and sex were consistently statistically significantly associated with MetS outcomes, indicated by MetS itself and both the binarized and continuous outcomes for the individual components (all *p* < 2.2 × 10^−16^), when adjusted for age. In addition, we noticed that differences across ethnicities were dependent on sex, indicated by a statistically significant interaction term for all outcomes, except the binary outcome high triglycerides (Fig. 1A).

Across all ethnicities, MetS occurred the most in participants from South-Asian Surinamese and Turkish descent in both sexes. However, the lowest prevalence of MetS was not specifically linked to one ethnicity. In females, the lowest prevalence was found in Dutch, while in males, MetS was least frequently observed in African Surinamese and Ghanaians. For the latter two ethnic groups, in contrast to the other ethnicities, MetS was not more prevalent in males than in females. In regard to the individual MetS components, generally reflected by both binarized and continuous outcomes, in both sexes, blood pressure was found to be higher in especially the Ghanaians, but also African Surinamese, whereas blood glucose and dyslipidemia was higher in South-Asian Surinamese, Turkish, and to a lesser extent Moroccans descent populations. For all ethnicities, these outcomes were in general higher in males compared to females. For obesity-related outcomes, again a clear sex-dependent difference across ethnicities was observed. Females in general had a higher prevalence of central obesity than males, but this difference was most pronounced in Ghanaians where females had the highest prevalence across ethnicities, while Ghanaian (and African Surinamese) males had the lowest prevalence across all ethnicities (Fig. 1A).

Taking socioeconomic status-, lifestyle-, and diet-related variables (known confounders for MetS) into account, both ethnicity (all *p* < 2.2 × 10^−16^) and sex (all *p* < 3.5 × 10^−12^) remained significant predictors for all outcomes. In addition, we noticed in general a similar pattern across the ethnicities and sexes as well as the sex-dependent differences across ethnicities (Additional file 1: Fig. S1).

### Combined metabolic risk patterns are heterogeneous across ethnicities

Different combinations of individual (binarized) MetS components potentially pose different risks for developing CVD. Prevalence of such combinations was also significantly different across ethnicities, both in males and in females, as well as in the subset of the participants with MetS (chi-square test, all *p* < 2.2 × 10^−16^) (Fig. 1B, C). For example, the healthiest combination (i.e., absence of all MetS components) occurred most often in the Dutch compared to the other ethnicities (Fig. 1B), both in males and in females. When focusing on the subset of subjects with MetS (Fig. 1C), males most often had the combination of central obesity, high blood pressure, and dysglycemia (WBG), as shown in the leftmost part of Fig. 1C. However, prevalence of this combination was highly different across ethnicities, with an especially high occurrence in Ghanaians. In women with MetS, either the same WBG combination (Dutch and Ghanaian) or the combination central obesity, high blood pressure, and low HDL (WBH) was most common (Turkish and Moroccan). In South-Asian and African Surinamese females, both WBG and WBH combinations had similar prevalence. The least healthy combination (i.e., presence of all MetS components together) was highest in Turkish males within the male population with MetS compared to the other ethnicities, while in the female population with MetS, this was highest in Dutch females. For both sexes, this prevalence was lowest in Ghanaians.

### Lower α-diversity is associated with worse metabolic outcomes in the total population

**Fig. 2:**
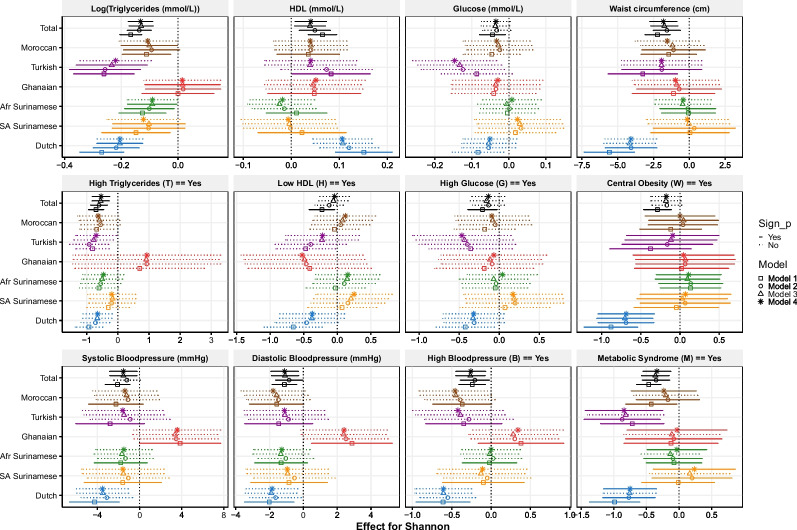
Overview of the effects of the Shannon index with 95% CI and *p*-values in the (logistic) regression models on MetS outcomes. For each model, each outcome measure was predicted with Shannon, sex, ethnicity (Dutch as reference), and sex:ethnicity (except high triglycerides) and additional covariates. Those models represent the ethnic-independent effect (i.e., total model). In addition, the effect per ethnicity is provided, which is derived from the model with an additional interaction term between ethnicity and Shannon. Significance (*p* < 0.05; Sign_p) of this overall interaction term, assessed via LRT, is indicated by line type, as well as the significance of the overall effect of the Shannon index in the total model. Analyses were performed on the subcohort (*n* = 3443) with microbiota data. For the binarized variables, logistic regression was performed and its effect is indicated by LogOdds ratio, while the others were analyzed with a linear regression model and their effect is indicated by the coefficients in the model. Effects per ethnicity were calculated based on the coefficients and standard errors obtained from the int model output, including the coefficients and variance–covariance matrix. Covariates included in models: model 1: age; model 2: model 1 + PPI use + socioeconomic status; model 3: model 2 + lifestyle; model 4: model 3 + diet

The gut microbiota composition has previously been shown to be associated with MetS and its individual components [15, 32], but this composition is different across ethnicities [12]. We hence used a subset of our cohort (*n* = 3443; characteristics displayed in Additional file 2: Table S1) to study associations between the gut microbiota composition and MetS and its binarized and continuous individual components. In regard to α-diversity, when we assumed the same effect across ethnicities on MetS outcomes, a statistically significant lower α-diversity was associated with worse MetS outcomes after adjusting for age, sex, and ethnicity (including the interaction term between sex and ethnicity if necessary, i.e., baseline model) (Additional file 3: Table S2). This was consistent for all outcomes, both binarized and continuous, when the Shannon index (combining evenness and richness) and Faith’s phylogenetic diversity (FaithPD) (a measure for phylogenic diversity; not for the binarized version of glucose) were considered. In models additively adding socioeconomic status (model 2)-, lifestyle (model 3)-, and diet (model 4)-related variables, the directions of associations between α-diversity and the MetS outcomes remained the same, although the effect size was often attenuated. Furthermore, statistically significant associations remained significant for all outcomes in both α-diversity indicators, except for glucose, DBP (only FaithPD), and the binarized versions of HDL, central obesity, and glucose (Fig. 2 and Additional file 1: Fig. S2). A statistically significant lower richness was only consistently observed for both triglyceride outcomes as well as for MetS itself and continuous HDL and waist circumference outcomes (Additional file 1: Fig. S3), which, except for MetS, remained statistically significant after adjusting for the socioeconomic status-, lifestyle-, and diet-related variables. Thus, in general, a lower α-diversity was associated with worse MetS outcomes when an ethnic-independent effect of α-diversity was assumed, sometimes even after adjusting for socioeconomic status-, lifestyle-, and diet-related variables, especially for triglycerides.

### Divergent associations of α-diversity with metabolic outcomes across ethnicities

Even though an ethnic-independent effect of α-diversity was statistically significant, the addition of an interaction term in the baseline model (i.e., model 1) showed that the association of α-diversity, represented by the Shannon index, and MetS differed across ethnicities for most continuous components (except for glucose) and MetS itself (Fig. 2, Additional file 3: Table S2). In Dutch, who have the highest α-diversity in general (Additional file 1: Fig. S4), a higher α-diversity was significantly associated with better MetS outcomes in regard to all components, but this was not always the case for all other ethnicities, although the direction of the effect was often similar. An aberrant opposing pattern was observed for Ghanaians in relation to blood pressure outcomes and triglycerides. In contrast to the other ethnicities, the Shannon index was significantly positively associated with blood pressure, and no significant association was found with triglycerides. Although the overall significant interaction between all ethnicities and α-diversity did not remain statistically significant after the addition of socioeconomic status-, lifestyle-, and diet-related variables for most outcomes (except for MetS itself) (Fig. 2), Ghanaians still had a significantly different association between the Shannon index and the previously mentioned MetS outcomes compared to the Dutch reference group. Furthermore, the general patterns across ethnicities remained similar.

Overall, statistically significant interactions between α-diversity and ethnicity were less frequently observed for the binarized versions and for the other α-diversity measures, but if significant (mainly central obesity and blood pressure related), often with the same patterns as for the Shannon index (Fig. 2, Additional file 1: Fig. S2 and S3, Additional file 3: Table S2).

### Several ASVs are robustly associated with metabolic indicators

**Fig. 3:**
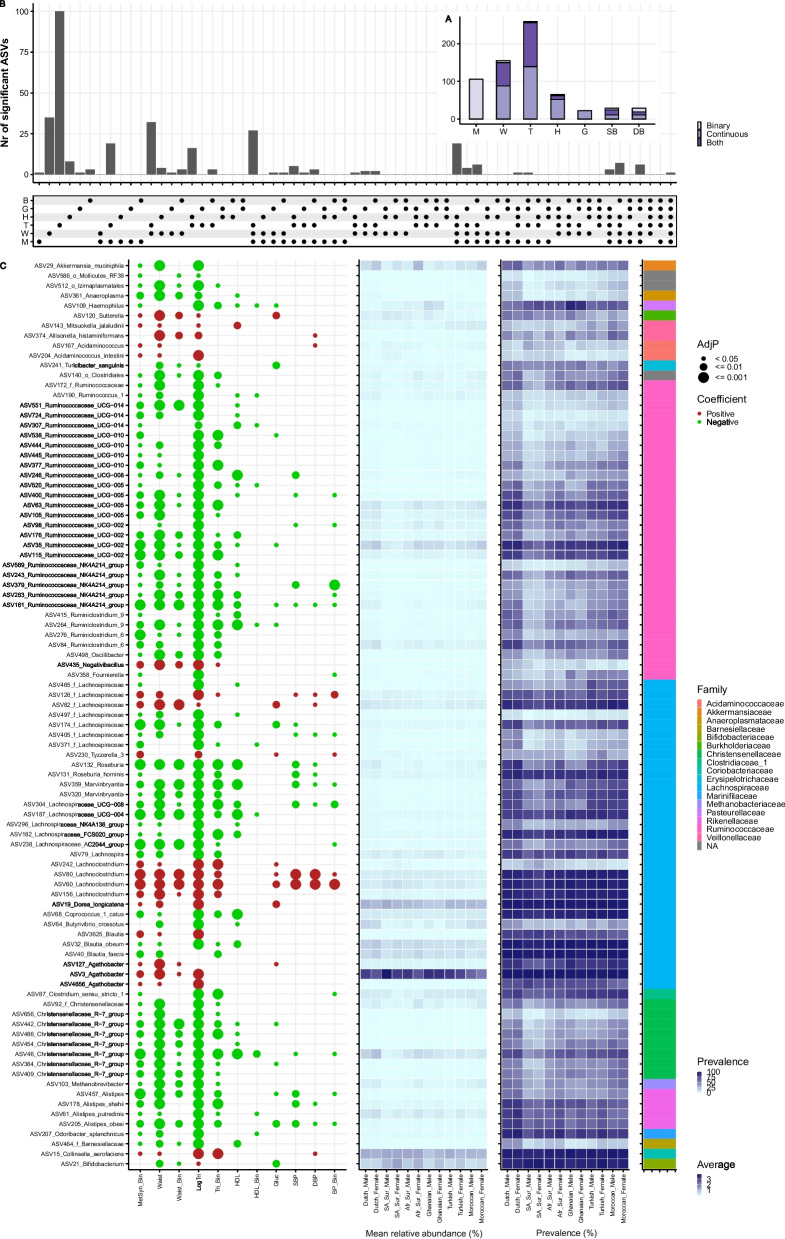
Overview of the (ethnic-independent) individual ASV analysis per MetS-related outcome (dependent variable), using (logistic) regression models. Models were run with the arcsin squared-root transformed ASV abundance as an independent variable and adjusted for age, sex, ethnicity (Dutch as reference), and sex:ethnicity (except for HighTri). Models and FDR correction was applied per outcome (either binarized or continuous). Analyses were performed on the subcohort (*n* = 3443) with microbiota data. **A** Overview of the number of significant ASVs (FDR corrected *p* < 0.05) per outcome (either binarized or continuous). Color indicates if the ASV is significant only for the continuous outcome, only for the binarized outcome or for both. For both SBP and DBP, High Bloodpressure =  = Yes is used as binarized outcome. **B** Overview of the number of significant ASVs per grouping of components. Per component, ASVs were selected for the combined outcome if it was significant for the binarized and/or continuous outcome. For blood pressure, SBP and DBP are taken together. M = metabolic syndrome, W = waist circumference, B = blood pressure, H = HDL, T = (log transformed) triglycerides, G = glucose. **C** Overview of a subset of the significant ASVs that were significant for at least 3 components, using the combined indication from **B** and using MetS itself as a separate component. For HDL, the direction of association is inverted, to make it more consistent with a healthier phenotype. *p*-values, direction of coefficients, taxonomical family of the ASV, and the mean relative abundance (%) and prevalence (%) are indicated per ASV

At the individual ASV level, ethnic-independent associations were also identified with MetS and all its individual components in the baseline models (Fig. 3 and Additional file 4: Table S3), after correction for multiple comparison with FDR. Most statistically significant hits were identified for the continuous values of the components, mostly belonging to triglycerides, followed by waist circumference. Several ASVs showed a robust association pattern, indicated by the same direction of associations across multiple components and/or consistently being associated with both the binarized and continuous version of the component (Fig. 3A, B). In the subset of ASVs that were significant for at least 3 different components, a relatively small set of ASVs assigned to *Lachnoclostridium* and *Agathobacter* was associated with worse MetS outcomes, while a larger set of ASVs, commonly of the *Ruminococcaceae*, *Lachnospiraceae*, and *Christensenellaceae* families, was mostly associated with better MetS outcomes (Fig. 3C). Although the number of ASVs that were statistically significant for at least 3 different components reduced greatly in models additionally adjusted for socioeconomic status-, lifestyle-, and diet-related covariates (model 4), we observed the same pattern for the abovementioned families (Additional file 1: Fig. S5 and Additional file 5: Table S4).

### ASVs robustly associated with metabolic indicators belong to the RCM trophic network, which is negatively associated with MetS outcomes in the total population

During subsequent hierarchical clustering analysis on the subset of statistically significant ASVs in the baseline models (Fig. 3C), we recognized that several of the *Ruminococcaceae* and *Christensenellaceae* ASVs belonged to the *Ruminococcaceae*, *Christensenellaceae*, and *Methanobrevibacter* (RCM) trophic network, previously identified by others [32, 33] (Additional file 1: Fig. S6). Interestingly, around half of the ASVs belonging to this network remained significant after adjustment for socioeconomic status-, lifestyle-, and diet-related covariates (model 4) (Additional file 1: Fig. S5).

Analysis on the transformed summed abundance of all ASVs in this RCM trophic network showed that it was also consistently associated with better MetS outcomes if the effect was assumed to be similar across all ethnicities (Additional file 1: Fig. S7, Additional file 6: Table S5) in the baseline models (i.e., model 1) but, in general, also after adjusting for socioeconomic status (model 2), lifestyle (model 3), and diet (model 4) variables, although the effect size was slightly attenuated. Importantly, this cluster was also highly correlated with the Shannon index (Pearson correlation = 0.71).

### Effects of RCM on several MetS outcomes are ethnic-dependent

Only a small proportion of the tested individual ASVs had a statistically significantly different effect across ethnicities on metabolic outcomes in the baseline models after correction for multiple comparisons (FDR). Those were mainly related to central obesity and MetS itself (Additional file 1: Fig. S8). However, remarkably, several of those ASVs were part of the previously mentioned RCM trophic network. Subsequent analysis on the transformed summed RCM abundance showed that its effect on various of the MetS outcomes differed across ethnicities, indicated by statistically significant interaction terms, except for the binarized triglyceride component (Additional file 1: Fig. S7; Additional file 6: Table S5) in the baseline models. Similar as for the Shannon index, in Dutch, the association of higher abundance with better MetS outcomes was significant for all outcomes, and in the Ghanaians, the relation to SBP and DBP was positive again. In the South-Asian Surinamese, the RCM trophic network was not associated with any of the outcomes at all but also not very abundant (Additional file 1: Fig. S4), while in Turkish, Moroccan, and African Surinamese, it was significant for some of the outcomes, including the continuous version of triglycerides and waist circumference. Remarkably, hierarchically adding socioeconomic status-, lifestyle-, and diet-related variables to the baseline model did not affect the statistical significance or pattern of the overall interaction between ethnicity and the RCM trophic network for half of the MetS outcomes.

## Discussion

In this study, we explored the ethnic specific occurrence of MetS and its individual components in metabolically untreated individuals from six different ethnicities, living in Amsterdam (The Netherlands), as well as the association between the gut microbiota composition and the different MetS outcomes in a subset of those individuals. Therefore, this study contributes to the still ongoing debate if the same conclusions can be drawn across different ethnicities in regard to MetS definition, occurrence pattern, and the role of the gut microbiota.

We showed that both binary and continuous indicators of the MetS components, as well as the prevalence of certain combinations of components, showed differences across ethnicities and were often sex-dependent. In regard to the gut microbiota composition, a small number of ASVs was found to be associated with worse MetS outcomes. However, higher abundance of most other ASVs, as well as a higher α-diversity, and a higher abundance of the RCM trophic network (previously associated with low BMI, low triglyceride levels and positively with α-diversity [32–34]) were robustly associated with better MetS outcomes, when ethnic-independent effects were assumed and often even after adjustment for known confounders of MetS. This was especially true in regard to waist and triglyceride-related measures. However, statistically significant ethnic-specific effects of the gut microbiota were noticed on several outcomes for especially the Shannon index and the RCM cluster. Associations of higher α-diversity and higher RCM network abundance with better MetS outcomes were often significant in the Dutch, but not always in all other ethnicities, although the direction was often similar. However, in Ghanaians, the Shannon index and RCM cluster showed an aberrant positive relation with blood pressure outcomes as compared to the other ethnicities. Although statistically significant overall interactions between gut microbiota and ethnicities were often less (or not) significant after adjustment for known confounders of MetS, aberrant associations were still observed for Ghanaians compared to the Dutch for some outcomes and patterns across ethnicities remained similar.

A differential, often sex-dependent, prevalence of MetS, its components, and their combinations were observed across ethnicities. Subjects from African descent (especially Ghanaian, but also African Surinamese) had higher values for blood pressure on average, while South-Asian Surinamese, Turkish, and to a lesser extent Moroccan had higher MetS rates and in general fared worse in regard to lipid-related measures. Several other studies have similarly noted differences across ethnicities for MetS and/or its components, including studies performed on our cohort [35, 36]. Although direct comparisons across cohorts are often difficult due to different diagnostic and inclusion criteria, South-Asian Surinamese are often mentioned to be more dyslipidemic compared to Caucasian Europeans, while in African Americans, high blood pressure is more common and contradictory also low triglyceride levels [6, 37–39]. Others have similarly reported on the sex-dependent ethnic heterogeneity across African Surinamese, South-Asian Surinamese, and Europeans [40, 41], especially for central obesity and MetS. In addition, although ethnic differences were not investigated, differences in the prevalence of specific combinations within European countries [3] and sexes [42] were previously recognized, and it was suggested that the risk for mortality or CVD is combination-dependent [4, 43, 44]. This might indicate that the definition of MetS actually combines different types of metabolic dysfunction and that from a pathophysiologic point of view, MetS is not a homogeneous syndrome, as suggested by Guize et al. [43]. Alternatively, if MetS is a single syndrome, it could also imply that different components have different weights in regard to MetS, dependent on sex or ethnicity, as suggested by Gurka et al. [8]. They for example mention that triglycerides were less correlated with MetS in African Americans compared to Hispanics or European Americans. Whether the current diagnostic criteria, or specific combinations of factors, are equally effective across ethnicities and sexes in identifying patients at risk for T2D or CVD remains thus to be further investigated.

Analysis on both the α-diversity and individual ASV level showed that various gut microbiota indicators were robustly associated with multiple MetS components when an ethnic-independent effect was assumed. Several of these robustly associated ASVs belonged to the RCM trophic network, which was highly correlated with the α-diversity. Other studies, mainly with Caucasian subjects, frequently make the same connection of high α-diversity generally being negatively associated with MetS risk factors [14, 15, 32, 45–48], yet associations between specific taxa and MetS or its components are often less consistent across studies, although *Christensenellaceae* is often mentioned [48]. However, when regarding these reported taxa from a (trophic-network) cluster like approach, the similarities between studies become more apparent. For example, in the Finnish METSIM cohort, a similar cluster of co-occurring OTUs, represented by OTUs from *Christensenellaceae*, *Ruminococcaceae*, Tenericutes, and *Methanobrevibacter*, was identified and positively associated with glutamine, acetate, and polyunsaturated fatty acids but negatively with triglycerides, glycerol, and glycA [32]. A similar analysis on the supplemental data of a Korean cohort reveals the exact same RCM cluster to be correlated with these MetS components [49]. Other studies also mentioned negative associations between *Christensenellaceae*, *Ruminococcaceae*, *Methanobrevibacter*, and Tenericutes with MetS and/or its components [33, 46, 48, 50]. The study of Ruaud et al. (2020) shows that this cooccurrence between *Christensenellaceae* and *Methanobrevibacter* is functional rather than just due to shared environmental preferences [51]. The H_2_ that is produced by *Christensenellaceae* species by fermentation is used as a substrate for methanogenesis by *Methanobrevibacter* species, indicative of cross-feeding. Furthermore, they showed that *Methanobrevibacter smithii* shifted the metabolic output of *Christensenella minuta* towards more acetate and H_2_ production and less butyrate, which hypothetically might result in less energy availability for the host and an accompanying lower BMI. This is also consistent with the positive association with acetate observed in the METSIM cohort. Clustering of *Christensenellaceae*, often considered to be the hub in those networks, with other taxa might also be due to its capability to produce H_2_ and acetate by providing substrates for other hydrogenotrophs or butyrate producers, including several *Ruminococcaceae* and *Roseburia* [52]. A high abundance of this RCM cluster thus seems to be indicative of the presence of a highly diverse trophic-network that seems to be related to a metabolically healthy host phenotype of which many of the between species and between host metabolic interactions have yet to be fully understood. Further research is needed to understand the exact mechanisms, including the potential mediating role of a high fiber and protein diet, with which *Christensenellaceae* has also been associated [53]. Many species that are part of the RCM cluster are however currently still uncultured making functional characterization of the cluster a prolonged challenge.

In addition to the RCM cluster, we identified several other (clusters of) taxa that were related to multiple MetS components that have been found by others as well [14, 15, 32, 46, 48]. This might indicate a common mechanism that either protects from or could contribute to the development of (parts of) MetS. Asnicar et al. (2021) for example show that *Haemophilus parainfluenzae* and *Turicibacter sanguinis* were, similarly to our study, related to health [14]. Asnicar also found several other bacterial groups typically associated with the *Bacteroides(2)* enterotype, like *Flavonifractor plautii*, *Ruminococcus gnavus*, and several *Clostridia* to be part of the disease cluster, similar to many of the ASVs identified in our “risk cluster” such as *Flavonifractor plautii* and ASVs assigned to *Lachnoclostridium*, *Agathobacter*, *Sutterella*, *Tyzzerella_3*, and *Collinsella aerofaciens.*

The multi-ethnic HELIUS study made it possible to look at potential ethnic-specific associations between the gut microbiota composition and MetS and its components. Statistically significant interactions between ethnicity and the gut microbiota indicators were particularly profound in regard to the Shannon diversity index and the RCM trophic network. Especially for Ghanaians, we identified an aberrant positive relationship with those indicators and blood pressure in the baseline models. While the overall significance of the interaction across ethnicities was not statistically significant anymore for the Shannon index after adjusting for additional confounders, the Ghanaians still had a significantly different effect size compared to the Dutch population. We can only speculate about the mechanisms behind these observations. We theorize that this aberrant association with hypertension might in part be linked with the fact that population of African descent are more salt-sensitive [54] and therefore could have a different etiology of hypertension. However, another study performed in 655 participants from Ghana, South Africa, Jamaica, and the USA with African ancestry did show a negative association between the Shannon index and hypertension in participants from Ghana and South Africa [45]. We similarly did not observe this same pattern in African Surinamese hinting that more factors than just genetics may be of importance including environmental ones. It could be that we have missed important confounders to include in our models, that the current confounders are not representative enough, or that the relationship between the current confounders and the MetS outcomes are not as important for the Ghanaians. Further research may shed light on those potential explanations.

Apart from the aberrant blood pressure pattern, statistically significantly different associations between ethnicity and α-diversity and/or the RCM trophic network abundance were also shown for other MetS components and MetS itself. Although not always explicitly tested for an interaction effect, other studies also mention potential ethnic differences in associations between the gut microbiome and, especially, central obesity-related measures. In two small studies comparing either African Americans or East Asians to European Americans, it was suggested that low α-diversity was more consistently related to high BMI in European Americans [55, 56]. Furthermore, the relation between *Christensenellaceae* and waist circumference might apply only to specific populations, as it was significantly associated in a Danish cohort, but not in an equally sized South Indian cohort [57]. We do not yet understand the mechanisms behind these ethnic discrepancies. It might be that in some ethnicities this cluster does not have the right (dietary) environment, by either missing important input metabolites or that important (intermediate) metabolites produced within this cluster are converted by other species into less beneficial metabolites. Since we did notice that the statistically significance of the overall interaction between ethnicity and the gut microbiota, in particular the Shannon index, on MetS outcomes was often not preserved after correction for known confounders of MetS (i.e., socioeconomic status-, lifestyle-, and diet-related variables), those confounders might indeed partly explain the observed differences. However, this could also be due to a lack of power since the Dutch constituted around a third of the total population with microbiota data. Genetic difference between ethnicities is bound to play a role but microbial compositional differences, such as a very low of abundance of the RCM trophic network as was here observed in South-Asian Surinamese, could in addition be behind some of the differential responses. Lastly, it is possible that this is a reflection of MetS heterogeneity as was also observed in the total cohort. These considerations are relevant, as more research is being focused on treatments aimed at altering the gut microbiome, for example fecal microbial transplants (FMTs) and/or simply dietary interventions. This might indicate that treatment needs to be tailored for each ethnicity individually. Additionally, the conclusions that are drawn from cohorts of European descent may not hold true for other populations, which is of importance considering that the vast majority of clinical trials are conducted on majority European descent cohorts.

Our study has several unique strengths. We included multiple ethnic minorities living in the same geographic area with a comparatively large sample size, including ethnicities that are rarely studied. Furthermore, we combined different levels of gut microbiota analyses (both summary statistics and individual ASVs) that were linked to each other and allowed us to look at it from a more holistic point of view. In addition, we analyzed both MetS itself as well as all its individual components (both binarized and continuous outcomes) that are part of the MetS definition. Lastly, instead of running the analyses separate per ethnicity, we included interaction terms in order to preserve power. In terms of limitations, having unequal sample sizes per ethnicity, especially in regard to the microbiota data, is not ideal as this might have resulted in a bias towards associations in the Dutch in the ethnic-independent analyses or that the number of interactions was underestimated. Furthermore, while different effects of the microbial composition were identified, we did not look at the potential function of the microbiome. As several different bacteria can have the same functionality, it could be that the relations at the functional level might be either more similar or even more divergent. As is the case with all cross-sectional studies, causal conclusions cannot be made. Since socioeconomic status, lifestyle, and diet could influence both the gut microbiota and the MetS outcomes considered here, we investigated their effect in additional models. However, those indicators, especially the diet-related indicators, are just proxies of those constructs, which potentially did not adequately capture the influence of those factors on the MetS outcomes. Indeed, as ethnicity is a complex construct, and might partly cover these factors, it is difficult to truly separate those effects from ethnic specific effects. Lastly, we included a relatively healthy study population by excluding participants on medication relating to MetS, which might have introduced some bias as exclusion due to medication usage was not equal across ethnicities. However, this also prevented potential confounders obfuscating results, as metformin for example is known to affect the gut microbiome composition [58].

## Conclusions

In conclusion, we showed that the prevalence of MetS itself, its individual components, and combinations thereof are different across ethnicities and are often sex-dependent. Furthermore, gut microbiota composition indicators (i.e., α-diversity, individual ASVs and the RCM trophic network), which differ across ethnicities, are mostly associated with better MetS outcomes if an ethnic-independent effect is assumed. However, statistically significant ethnic-dependent associations with MetS outcomes were observed for α-diversity and the RCM trophic network. In particular, a higher diversity was significantly associated with better MetS outcomes in Dutch and sometimes other ethnicities, whereas in Ghanaians, it associated with high blood pressure outcomes. Even though adjustment for socioeconomic status-, lifestyle-, and diet-related variables often attenuated the effect size and/or the statistical significance of the ethnic-specific associations, an overall similar pattern across outcomes and ethnicities remained. These findings highlight the complex heterogeneous nature of MetS itself and the need for more research in its occurrence and effectiveness in different ethnicities as well as the potential contribution of the gut microbiota to this disease.

### Supplementary Information

**Additional file 1: Fig S1-S8.** PDF file with all supplementary figures S1-S8 and corresponding figure legends.

**Additional file 2: Table S1.** Population characteristics for the subset of the cohort with gut microbiota data. Overview of population characteristics for the Dutch, South-Asian Surinamese (SA Surinamese), African Surinamese (Afr Surinamese), Ghanaian, Turkish and Moroccan, presented separately per sex.

**Additional file 3: Table S2.** Overview of the effects for the α-diversity indices with 95% CI and p-values in the (logistic) regression models on MetS outcomes. For each model type, each outcome measure was predicted with an AlphaIndicator (Shannon index, Richness or Faith’s PD), sex, ethnicity (Dutch as reference), (except HighTri) sex:ethnicity and additional covariates (model_total, representing ethnic-independent effects). In a follow-up model, an interaction term between ethnicity and the AlphaIndicator (int model; effect for ethnicities) was added to the total model, and overall significance was assessed with a LRT. Analyses were performed on the subcohort (*n* = 3443) with microbiota data. Logistic regression was performed for variables indicated with the term Binarized and the effect is indicated as a LogOdds ratio, while the others were analyzed with a linear regression model where effect is indicated by the coefficients in the model. Effects per ethnicity were calculated based on the coefficients and standard errors obtained from the int model output, including the coefficients and variance-covariance matrix. Covariates included in models: model 1: age; model 2: model 1 + PPI use + socioeconomic status; model 3: model 2 + lifestyle; model 4: model 3 + diet.

**Additional file 4: Table S3.** Overview of the effects and (adjusted) *p*-values for the ASVs in the (logistic) regression models on MetS outcomes. Each outcome measure was predicted with the arcsin squared-root transformed relative ASV abundance, sex, ethnicity (Dutch as reference), age and (except HighTri) sex:ethnicity (ethnic-independent model; effect and p-values provided) and with an additional interaction term between ethnicity and ASV (int_eth model; only *p*-values provided, assessed with LRT). Analyses were performed on the subcohort (*n* = 3443) with microbiota data. Logistic regression was performed for variables indicated with “Bin_” and the effect is indicated as a LogOdds ratio, while the others were analyzed with a linear regression model and where the effect is indicated by the coefficients in the model. Per outcome, and per model (with or without interaction term), the analyses were corrected for multiple comparisons with the Benjamini-Hochberg correction. Those *p*-values are indicated with “adj_p.”

**Additional file 5: Table S4.** Overview of the effects and (adjusted) p-values for the ASVs in the (logistic) regression models on MetS outcomes. Each outcome measure was predicted with the arcsin squared-root transformed relative ASV abundance, sex, ethnicity (Dutch as reference), age, PPI use, socioeconomic status, lifestyle, diet and (except HighTri) sex:ethnicity (ethnic-independent model; effect and p-values provided) and with an additional interaction term between ethnicity and ASV (int_eth model; only p-values provided, assessed with LRT). Analyses were performed on the subcohort (*n* = 3443) with microbiota data. Logistic regression was performed for variables indicated with “Bin_” and the effect is indicated as a LogOdds ratio, while the others were analyzed with a linear regression model and where the effect is indicated by the coefficients in the model. Per outcome, and per model (with or without interaction term), the analyses were corrected for multiple comparisons with the Benjamini-Hochberg correction. Those p-values are indicated with “adj_p.”

**Additional file 6: Table S5.** Overview of the effects for the RCM cluster with 95% CI and p-values in the (logistic) regression models on MetS outcomes. For each model type, each outcome measure was predicted with the arcsin squared-root transformed summed relative abundances of ASVs belonging to the RCM cluster, sex, ethnicity (Dutch as reference), (except HighTri) sex:ethnicity and additional covariates (model_total, representing ethnic-independent effects). In a follow-up model, an interaction term between ethnicity and the RCM cluster (int model; effect for ethnicities derived from the covariance matrix and the model output) was added to the total model, and overall significance was assessed with a LRT. Analyses were performed on the subcohort (*n* = 3443) with microbiota data. Logistic regression was performed for variables indicated with the term Binarized and the effect is indicated as a LogOdds ratio, while the others were analyzed with a linear regression model where effect is indicated by the coefficients in the model. Effects per ethnicity were calculated based on the coefficients and standard errors obtained from the int model output, including the coefficients and variance-covariance matrix. Covariates included in models: model 1: age; model 2: model 1 + PPI use + socioeconomic status; model 3: model 2 + lifestyle; model 4: model 3 + diet.

**Additional file 7.** Example of the data transfer agreement necessary to obtain the microbiota HELIUS data.

## Data Availability

The HELIUS data are owned by the Amsterdam University Medical Centers, location AMC, in Amsterdam, the Netherlands. The raw 16S sequencing data from all samples that have been sequenced have been deposited in the European Genome-Phenome Archive database under accession code EGAD00001004106 (https://ega-archive.org/datasets/EGAD00001004106) [18]. Because of restrictions imposed by the signed consent of the participants, the sequencing data are only available under restricted access. Access is granted to all researchers that are affiliated with an internationally recognized research institution who request the HELIUS data within the EGA context and have signed the data transfer agreement (Additional file 7). Any researcher can request the data (including the metadata and used ASV table) by submitting a proposal to the HELIUS Executive Board as outlined at http://www.heliusstudy.nl/en/researchers/collaboration by email: heliuscoordinator@amsterdamumc.nl. The HELIUS Executive Board will check proposals for compatibility with the general objectives, ethical approvals, and informed consent forms of the HELIUS study. Requests submitted to the HELIUS Executive Board are evaluated in principle within 3 weeks. There are no other restrictions to obtain the data, and all data requests will be processed in the same manner.

## References

[1] Virani SS (2021). Heart disease and stroke statistics - 2021 update: a report from the American Heart Association. Circulation..

[2] Alberti KGMM (2009). Harmonizing the metabolic syndrome: a joint interim statement of the International Diabetes Federation Task Force on Epidemiology and Prevention; National Heart, Lung, and Blood Institute; American Heart Association; World heart federation. International Circulation.

[3] Scuteri A (2015). Metabolic syndrome across Europe: different clusters of risk factors. Eur J Prev Cardiol.

[4] Franco OH (2009). Trajectories of entering the metabolic syndrome: the Framingham Heart study. Circulation.

[5] Ostchega Y, Fryar CD, Nwankwo T, Nguyen DT. Hypertension prevalence among adults aged 18 and over: United States, 2017–2018. NCHS Data Brief. 2020;364:1–8. 32487290

[6] Frank ATH (2014). Racial/ethnic differences in dyslipidemia patterns. Circulation.

[7] Sumner AE, Cowie CC (2008). Ethnic differences in the ability of triglyceride levels to identify insulin resistance. Atherosclerosis.

[8] Gurka MJ, Lilly CL, Oliver MN, Deboer MD (2014). An examination of sex and racial/ethnic differences in the metabolic syndrome among adults: a confirmatory factor analysis and a resulting continuous severity score. Metabolism.

[9] Burchard EG (2003). The importance of race and ethnic background in biomedical research and clinical practice. N Engl J Med.

[10] Rao S (2021). Association of genetic West African ancestry, blood pressure response to therapy, and cardiovascular risk among self-reported Black individuals in the Systolic Blood Pressure Reduction Intervention Trial (SPRINT). JAMA Cardiol.

[11] Mensah GA (2019). Emerging concepts in precision medicine and cardiovascular diseases in racial and ethnic minority populations. Circ Res.

[12] Deschasaux M (2018). Depicting the composition of gut microbiota in a population with varied ethnic origins but shared geography. Nat Med.

[13] Kootte RS (2017). Improvement of insulin sensitivity after lean donor feces in metabolic syndrome is driven by baseline intestinal microbiota composition. Cell Metab.

[14] Asnicar F (2021). Microbiome connections with host metabolism and habitual diet from 1,098 deeply phenotyped individuals. Nat Med.

[15] Walker RL (2021). Population study of the gut microbiome: associations with diet, lifestyle, and cardiometabolic disease. Genome Med.

[16] Snijder MB (2017). Cohort profile: the Healthy Life in an Urban Setting (HELIUS) study in Amsterdam, the Netherlands. BMJ Open.

[17] Stronks K (2013). Unravelling the impact of ethnicity on health in Europe: the HELIUS study. BMC Public Health.

[18] Verhaar BJH, et al. Associations between gut microbiota, faecal short-chain fatty acids, and blood pressure across ethnic groups: the HELIUS study. *EGAD00001004106, European Genome-phenome Archive*. 2020. https://ega-archive.org/datasets/EGAD00001004106. 10.1093/eurheartj/ehaa704 PMC7724641 32869053

[19] Perini W, Kunst AE, Snijder MB, Peters RJG, van Valkengoed IGM (2019). Ethnic differences in metabolic cardiovascular risk among normal weight individuals: implications for cardiovascular risk screening. The HELIUS study. Nutr Metab Cardiovasc Dis.

[20] Stronks K, Kulu-Glasgow I, Agyemang C (2009). The utility of ‘country of birth’ for the classification of ethnic groups in health research: the Dutch experience. Ethn Health.

[21] Balvers M (2021). Analyzing type 2 diabetes associations with the gut microbiome in individuals from two ethnic backgrounds living in the same geographic area. Nutrients.

[22] Edgar RC (2010). Search and clustering orders of magnitude faster than BLAST. Bioinformatics.

[23] Callahan BJ (2016). DADA2: High-resolution sample inference from Illumina amplicon data. Nat Methods.

[24] Quast C (2013). The SILVA ribosomal RNA gene database project: improved data processing and web-based tools. Nucleic Acids Res.

[25] Katoh K, Misawa K, Kuma KI, Miyata T (2002). MAFFT: A novel method for rapid multiple sequence alignment based on fast Fourier transform. Nucleic Acids Res.

[26] Katoh K, Standley DM (2013). MAFFT multiple sequence alignment software version 7: Improvements in performance and usability. Mol Biol Evol.

[27] Price MN, Dehal PS, Arkin AP (2010). FastTree 2 - approximately maximum-likelihood trees for large alignments. PLoS One.

[28] Oksanen J, Simpson GL, Guillaume Blanchet F, Kindt R, Legendre P, Minchin PR, et al. vegan: Community Ecology Package. R package version 2.6-4. 2022. https://CRAN.R-project.org/package=vegan.

[29] Kembel SW (2010). Picante: R tools for integrating phylogenies and ecology. Bioinformatics.

[30] Benjamini Y, Hochberg Y (1995). Controlling the false discovery rate: a practical and powerful approach to multiple testing. J R Stat Soc Ser B.

[31] Team RC (2020). R: a language and environment for statistical computing.

[32] Org E (2017). Relationships between gut microbiota, plasma metabolites, and metabolic syndrome traits in the METSIM cohort. Genome Biol.

[33] Li X (2020). Regional distribution of Christensenellaceae and its associations with metabolic syndrome based on a population-level analysis. PeerJ.

[34] Goodrich J (2014). Human genetics shape the gut microbiome. Cell.

[35] Gazzola K (2018). Ethnic differences in plasma lipid levels in a large multiethnic cohort: the HELIUS study. J Clin Lipidol.

[36] van Laer SD, Snijder MB, Agyemang C, Peters RJG, van den Born BJH (2018). Ethnic differences in hypertension prevalence and contributing determinants – the HELIUS study. Eur J Prev Cardiol.

[37] Tillin T (2013). The relationship between metabolic risk factors and incident cardiovascular disease in Europeans, South Asians, and African Caribbeans: SABRE (Southall and Brent Revisited) - a prospective population-based study. J Am Coll Cardiol.

[38] Almulhem M (2021). Cardio-metabolic outcomes in South Asians compared to White Europeans in the United Kingdom: a matched controlled population-based cohort study. BMC Cardiovasc Disord.

[39] Bentley AR, Rotimi CN (2017). Interethnic differences in serum lipids and implications for cardiometabolic disease risk in African ancestry populations. Glob Heart.

[40] Agyemang C, van Valkengoed IG, van den Born BJ, Bhopal R, Stronks K (2012). Heterogeneity in sex differences in the metabolic syndrome in Dutch white, Surinamese African and South Asian populations. Diabet Med.

[41] Tillin T (2005). Metabolic syndrome and coronary heart disease in South Asians, African-Caribbeans and white Europeans: A UK population-based cross-sectional study. Diabetologia.

[42] Moebus S (2010). Age- and sex-specific prevalence and ten-year risk for cardiovascular disease of all 16 risk factor combinations of the metabolic syndrome - a cross-sectional study. Cardiovasc Diabetol.

[43] Guize L (2007). All-cause mortality associated with specific combinations of the metabolic syndrome according to recent definitions. Diabetes Care.

[44] Yu WW, Randhawa AK, Blair SN, Sui X, Kuk JL (2019). Age- and sex- specific all-cause mortality risk greatest in metabolic syndrome combinations with elevated blood pressure from 7 U.S. Cohorts. PLoS One.

[45] Fei N (2019). The human microbiota is associated with cardiometabolic risk across the epidemiologic transition. PLoS One.

[46] Fu J (2015). The gut microbiome contributes to a substantial proportion of the variation in blood lipids. Circ Res.

[47] Lim MY (2017). The effect of heritability and host genetics on the gut microbiota and metabolic syndrome. Gut.

[48] He Y (2018). Linking gut microbiota, metabolic syndrome and economic status based on a population-level analysis. Microbiome.

[49] Song JS, Kim JOR, Yoon SM, Kwon M-J, Ki C-S (2023). The association between gut microbiome and hypertension varies according to enterotypes: a Korean study. Front Microbiomes.

[50] Guzmán-Castañeda SJ (2020). Gut microbiota composition explains more variance in the host cardiometabolic risk than genetic ancestry. Gut Microbes.

[51] Ruaud A (2020). Syntrophy via Interspecies H2 transfer between Christensenella and Methanobrevibacter underlies their global cooccurence in the human gut. MBio.

[52] Louis P, Flint HJ (2017). Formation of propionate and butyrate by the human colonic microbiota. Environ Microbiol.

[53] Waters JL, Ley RE (2019). The human gut bacteria Christensenellaceae are widespread, heritable, and associated with health. BMC Biol.

[54] Morris RC, Sebastian A, Forman A, Tanaka M, Schmidlin O (1999). Normotensive salt sensitivity: effects of race and dietary potassium. Hypertension.

[55] Stanislawski MA, Dabelea D, Lange LA, Wagner BD, Lozupone CA (2019). Gut microbiota phenotypes of obesity. NPJ Biofilms Microbiomes..

[56] Ang QY (2021). The east asian gut microbiome is distinct from colocalized white subjects and connected to metabolic health. Elife.

[57] Alvarez-silva C (2021). Trans-ethnic gut microbiota signatures of type 2 diabetes in Denmark and India. Genome Med.

[58] Wu H (2017). Metformin alters the gut microbiome of individuals with treatment-naive type 2 diabetes, contributing to the therapeutic effects of the drug. Nat Med.

