# Supplementary material for: Ethnic variations in metabolic syndrome components and their associations with the gut microbiota: the HELIUS study
Source: Genome Med. 2024 Mar 20;16:41. doi: 10.1186/s13073-024-01295-7 (PMC10953122; doi:10.1186/s13073-024-01295-7)
Supplement: Supplementary file 1 — Additional file 1: Fig S1-S8. PDF file with all supplementary figures S1-S8 and corresponding figure legends. [file 13073_2024_1295_MOESM1_ESM.pdf]

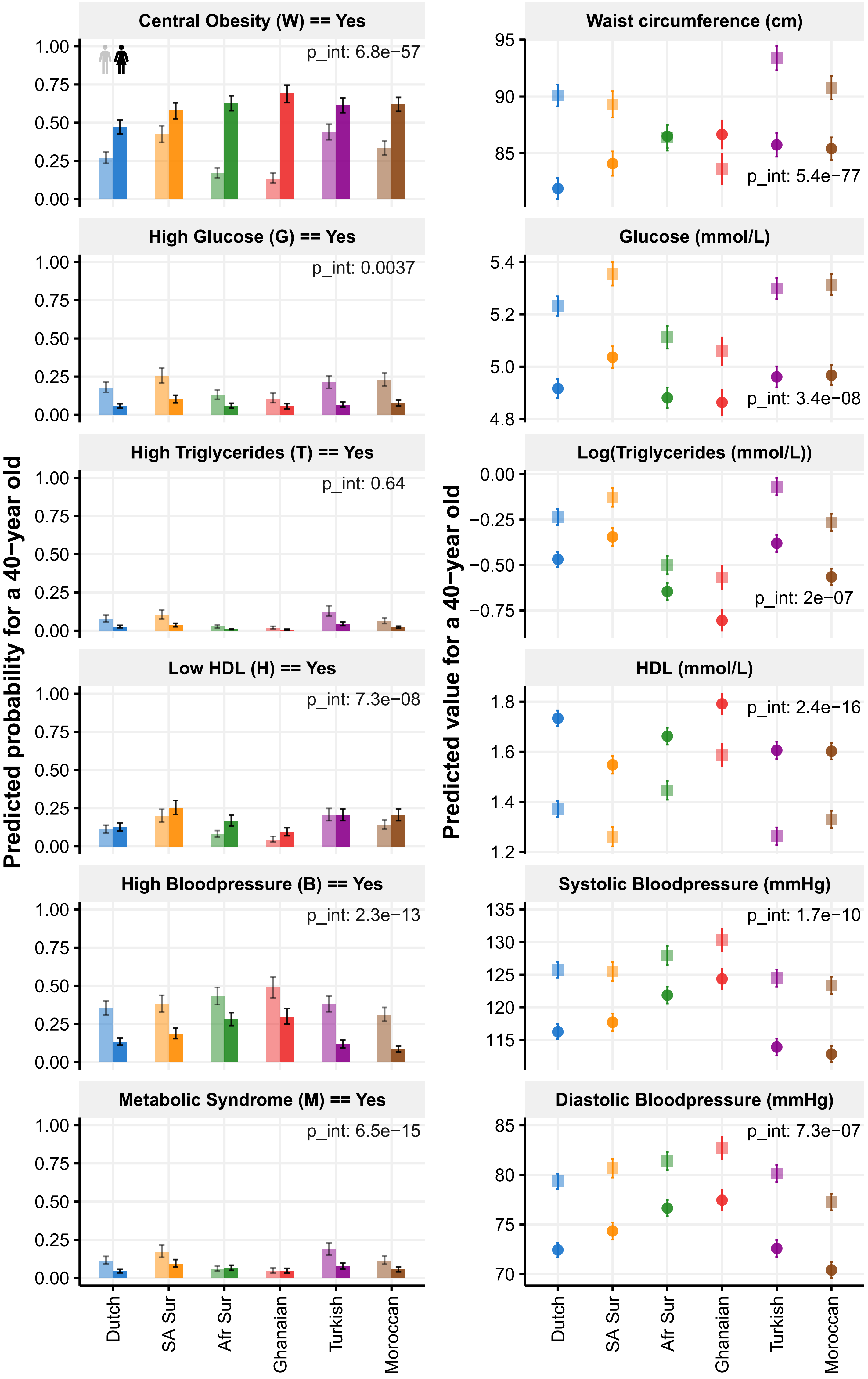

Fig. S1: Overview of the occurrence of the metabolic syndrome (MetS) related measures in the total population (n=16,209) after adjustment for socioeconomic, lifestyle and diet related variables. Predicted outcomes (with 95% CI) for the (logistic) regression models are shown with each outcome measure predicted on age, sex, ethnicity, socioeconomic, lifestyle, diet and (except for HighTri) sex:ethnicity. Values are provided for a 40 years old person, who worked, had an academic occupational level, higher educational level, did not smoke, was physically active, did not take alcohol, took regularly fruit, but did not take regularly sugar drinks, from the different groups. P-values for the interaction term (assessed via LRT) in the model are stated. The left column represents the binarized outcomes, the right column represents the continuous outcomes.

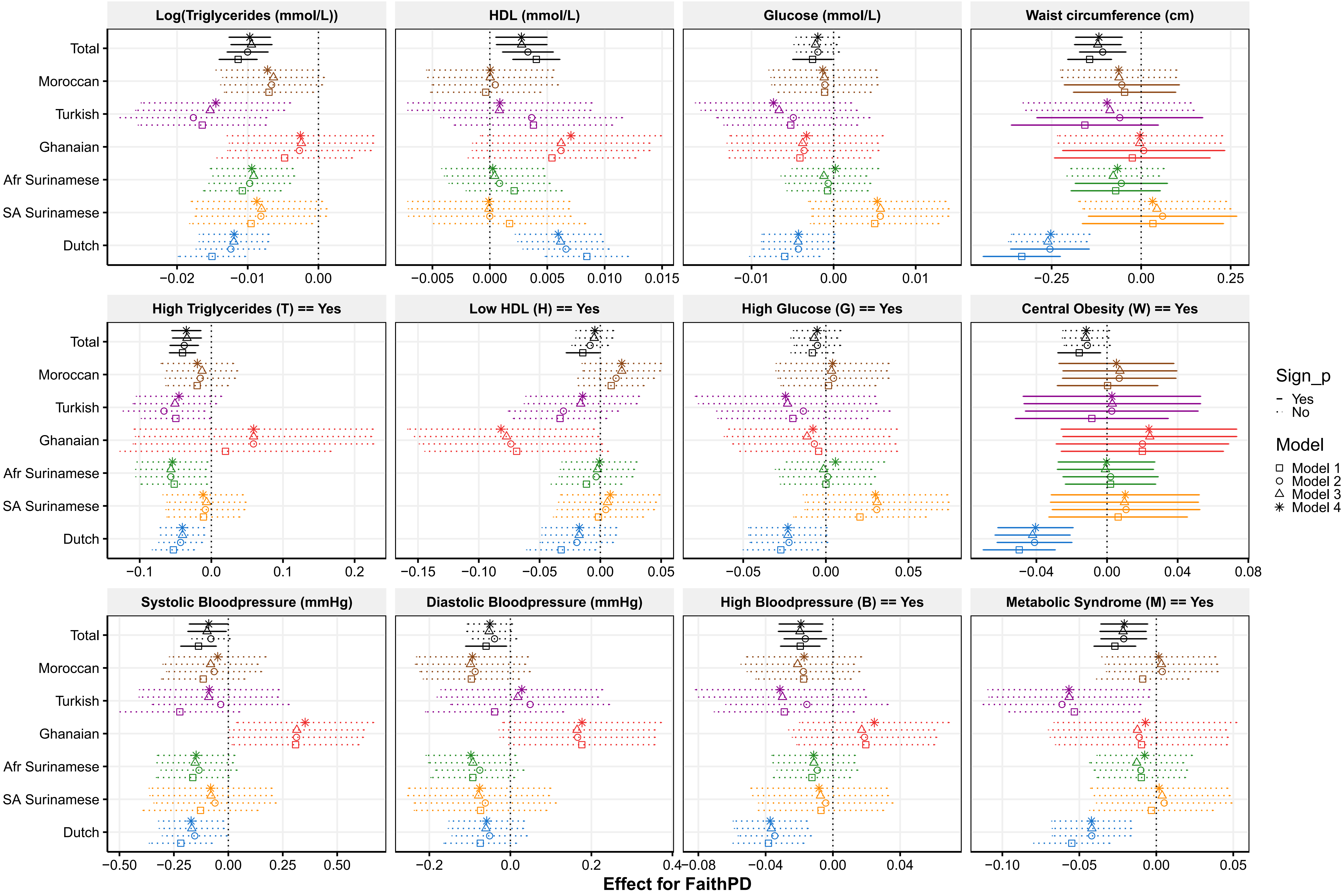

Fig. S2: Overview of the effects of Faith's PD with 95% CI and p-values in the (logistic) regression models on MetS outcomes. For each model, each outcome measure was predicted with Faith's PD, sex, ethnicity (Dutch as reference), and sex:ethnicity (except High Triglycerides) and additional covariates. Those models represent the ethnic-independent effect (i.e. Total model). In addition, the effect per ethnicity is provided, which is derived from the model with an additional interaction term between ethnicity and Faith's PD. Significance ($p < 0.05$; Sign_p) of this overall interaction term, assessed via LRT, is indicated by line type, as well as the significance of the overall effect of Faith's PD in the Total model. Analyses were performed on the subcohort (n=3443) with microbiota data. For the binarized variables, logistic regression was performed and its effect is indicated by LogOdds ratio, while the others were analysed with a linear regression model and their effect is indicated by the coefficients in the model. Effects per ethnicity were calculated based on the coefficients and standard errors obtained from the int model output, including the coefficients and variance-covariance matrix. Covariates included in models: model 1: age; model 2: model 1 + PPI use + socioeconomic status; model 3: model 2 + lifestyle; model 4: model 3 + diet.

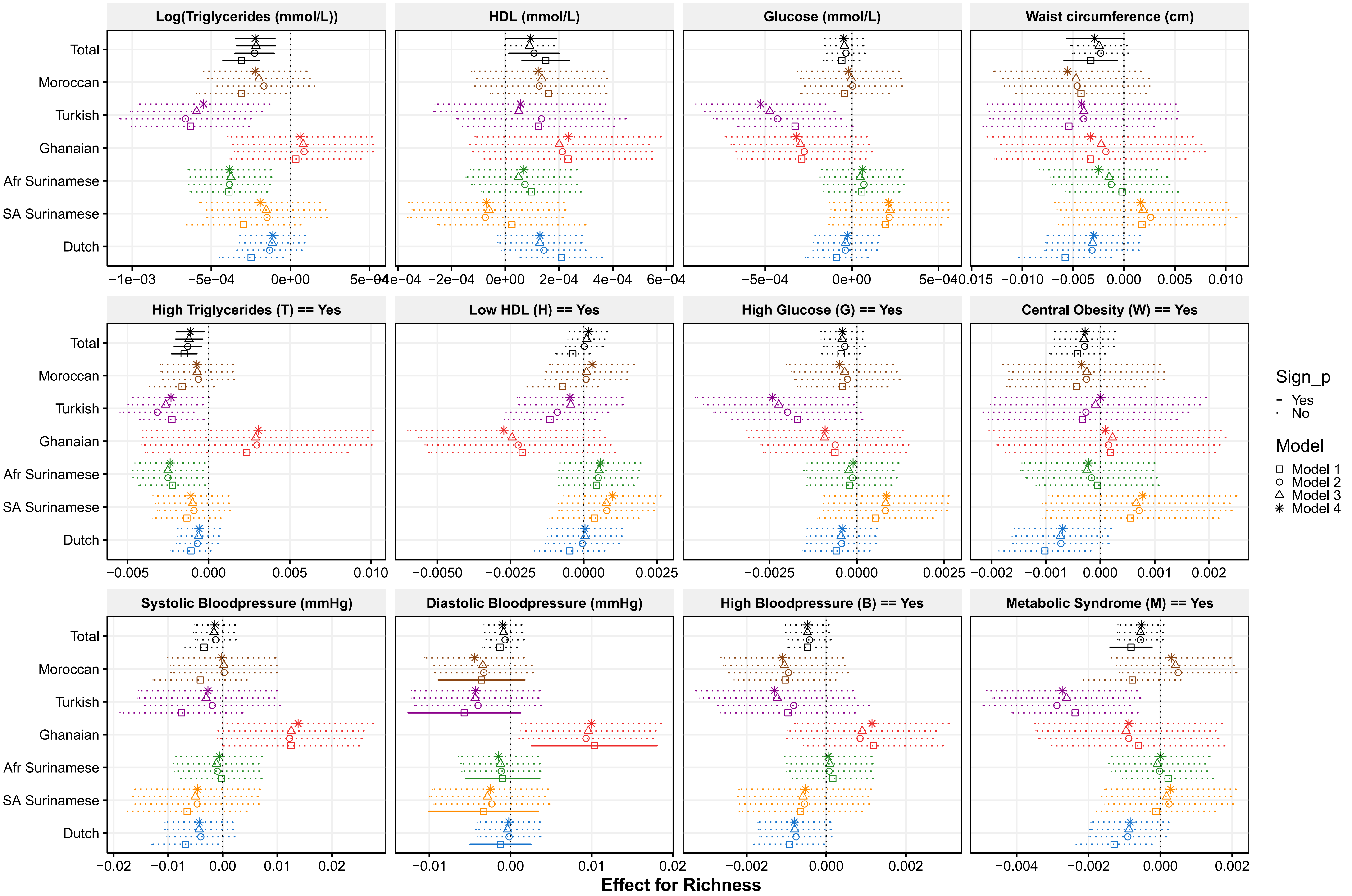

Fig. S3: Overview of the effects of Richness with 95% CI and p-values in the (logistic) regression models on MetS outcomes. For each model, each outcome measure was predicted with Richness, sex, ethnicity (Dutch as reference), and sex:ethnicity (except High Triglycerides) and additional covariates. Those models represent the ethnic-independent effect (i.e. Total model). In addition, the effect per ethnicity is provided, which is derived from the model with an additional interaction term between ethnicity and Richness. Significance ($p < 0.05$; Sign_p) of this overall interaction term, assessed via LRT, is indicated by line type, as well as the significance of the overall effect of Richness in the Total model. Analyses were performed on the subcohort (n=3443) with microbiota data. For the binarized variables, logistic regression was performed and its effect is indicated by LogOdds ratio, while the others were analysed with a linear regression model and their effect is indicated by the coefficients in the model. Effects per ethnicity were calculated based on the coefficients and standard errors obtained from the int model output, including the coefficients and variance-covariance matrix. Covariates included in models: model 1: age; model 2: model 1 + PPI use + socioeconomic status; model 3: model 2 + lifestyle; model 4: model 3 + diet.

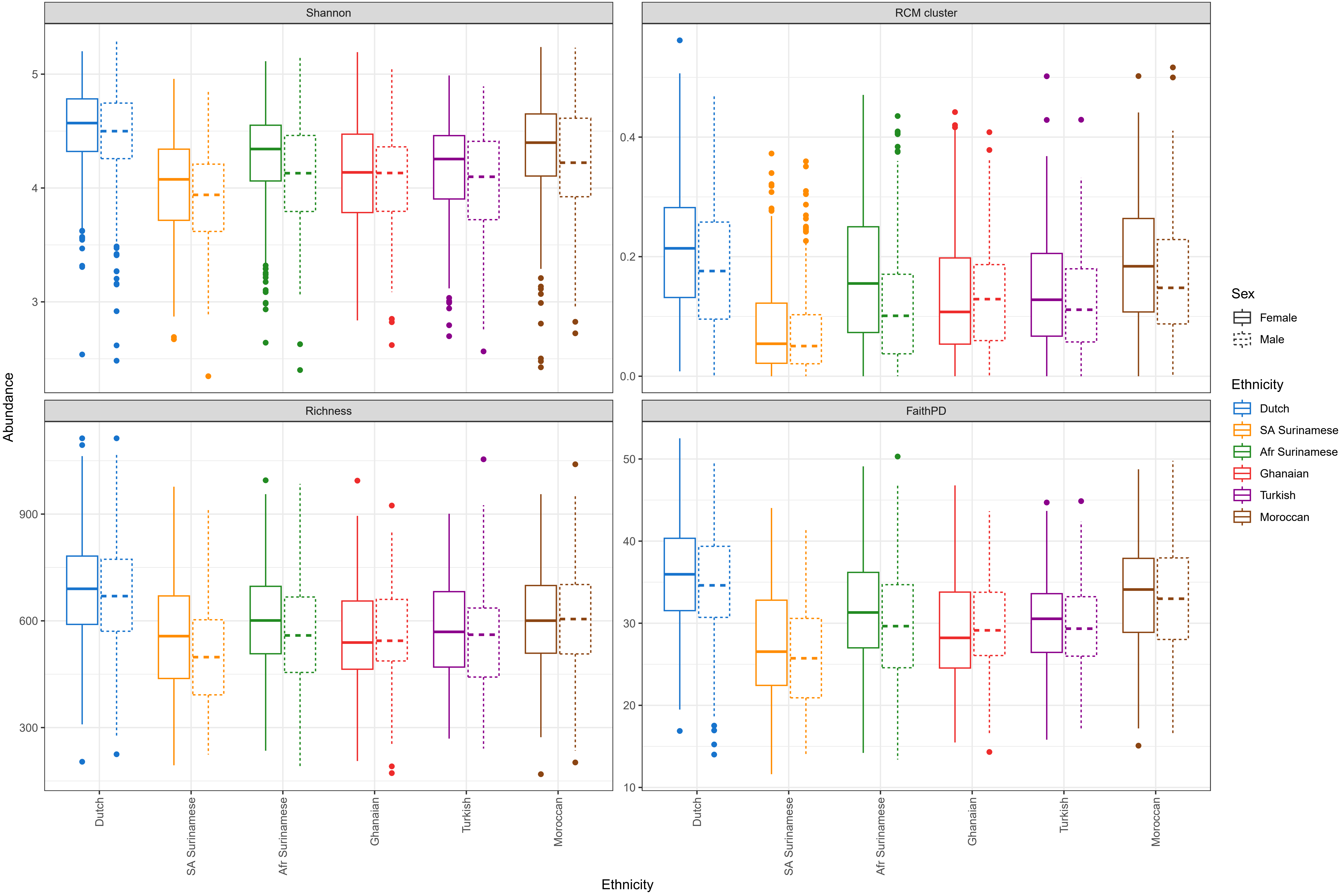

Fig. S4: Overview of the distribution of the 3 α-diversity indicators (Shannon index, Faith's PD and Richness) per ethnicity and sex as well as the arcsin squared-root transformed RCM network abundance.

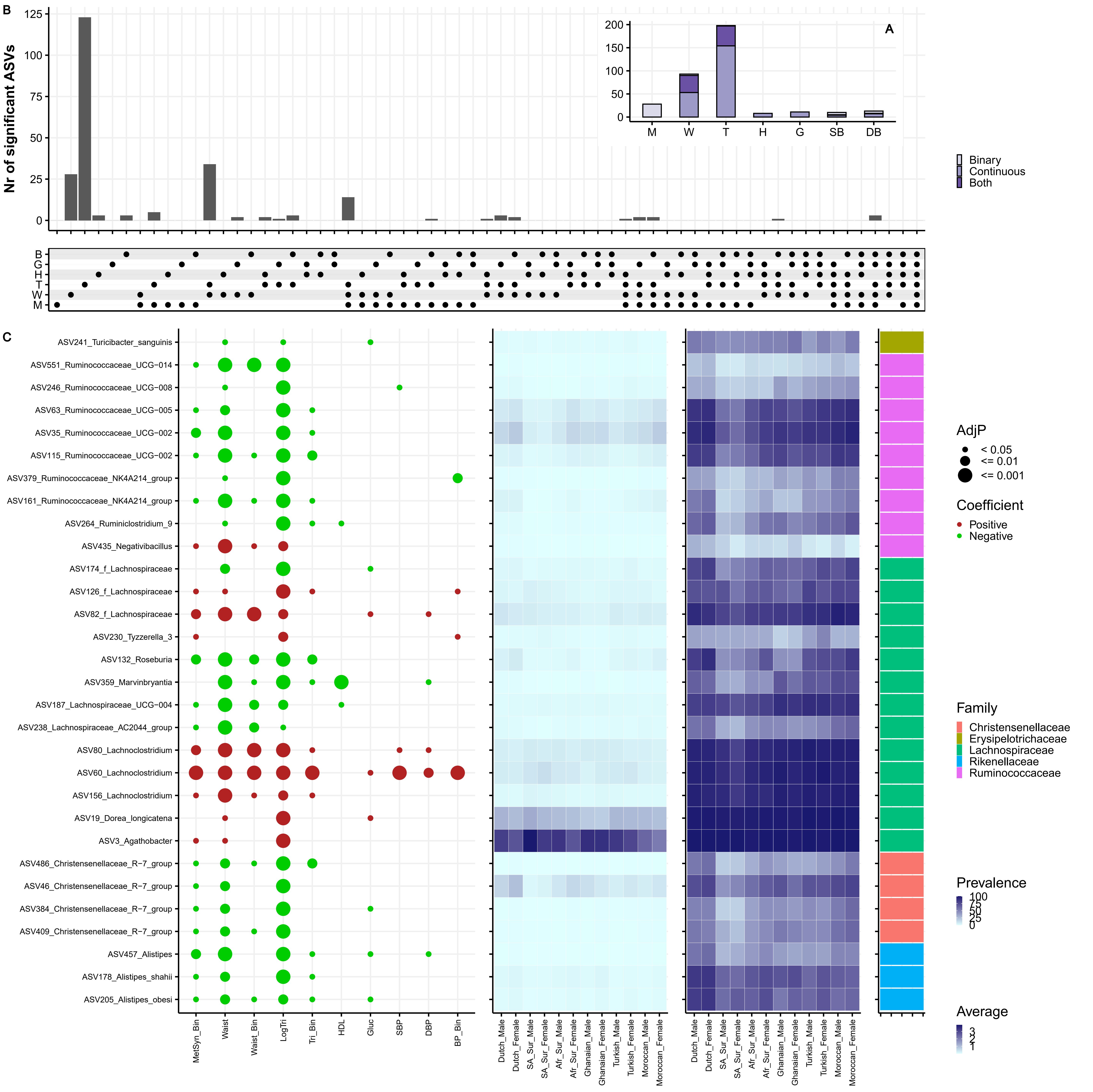

Fig. S5: Overview of the (ethnic-independent) individual ASV analysis per MetS related outcome (dependent variable), using (logistic) regression models. Models were run with the arcsin squared-root transformed ASV abundance as an independent variable and adjusted for age, sex, ethnicity (Dutch as reference), socioeconomic status, lifestyle, diet, PPI use and sex:ethnicity (except for HighTri). Models and FDR correction was applied per outcome (either binarized or continuous). Analyses were performed on the subcohort (n=3443) with microbiota data. A) Overview of the number of significant ASVs (FDR corrected p < 0.05) per outcome (either binarized or continuous). Color indicates if the ASV is significant only for the continuous outcome, only for the binarized outcome or for both. For both SBP and DBP, High Bloodpressure == Yes is used as binarized outcome. B) Overview of the number of significant ASVs per grouping of components. Per component, ASVs were selected for the combined outcome if it was significant for the binarized and/or continuous outcome. For blood pressure, SBP and DBP are taken together. M = Metabolic Syndrome, W = Waist circumference, B = Blood pressure, H = HDL, T = (log transformed) Triglycerides, G = Glucose. C) Overview of a subset of the significant ASVs that were significant for at least 3 components, using the combined indication from B and using MetS itself as a separate component. For HDL, the direction of association is inverted, to make it more consistent with a healthier phenotype. P-values, direction of coefficients, taxonomical family of the ASV and the mean relative abundance (%) and prevalence (%) is indicated per ASV.

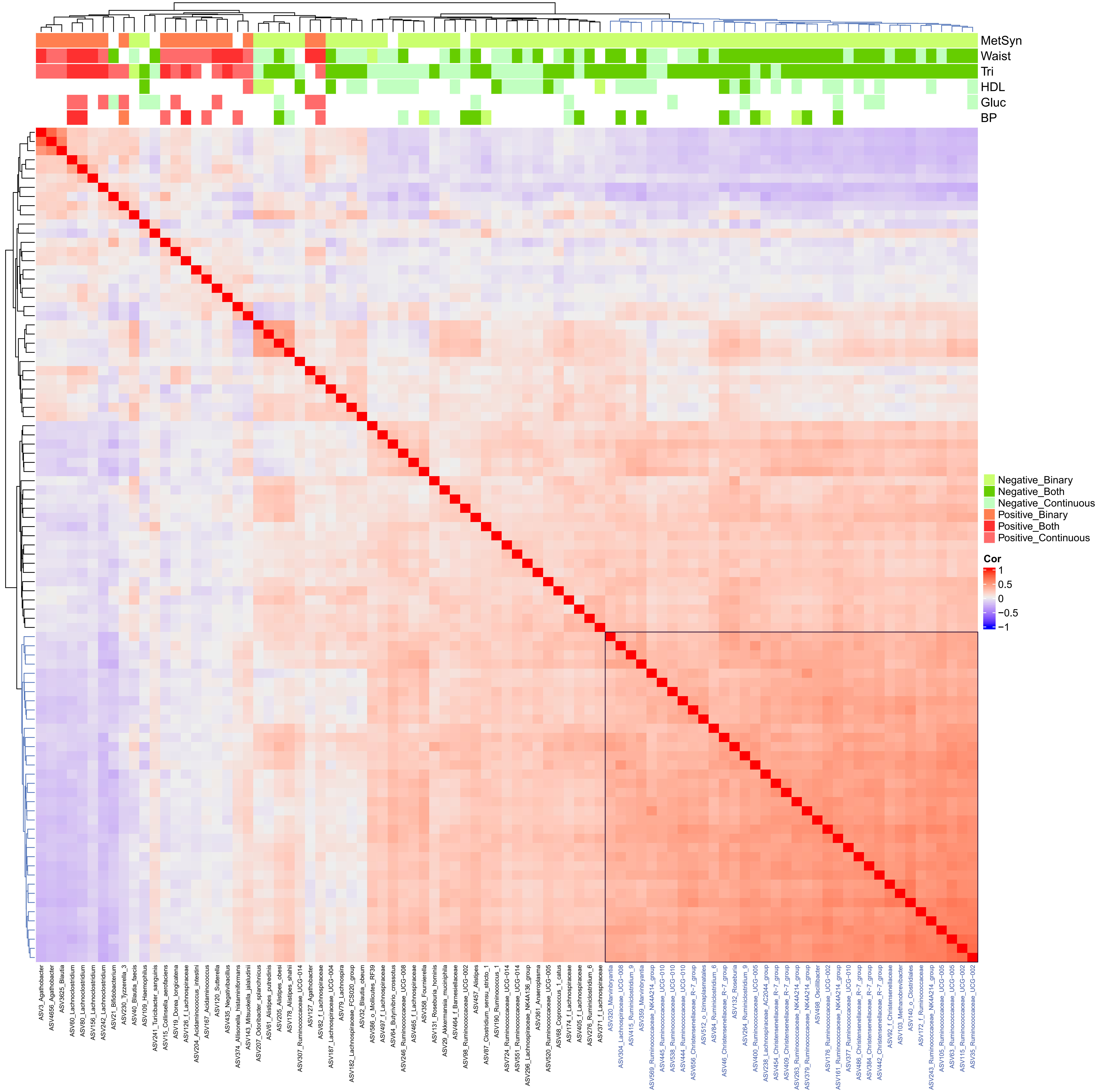

Fig. S6: Overview of the clustering of the ASVs that were significant for at least 3 components (including MetS), using hierarchical clustering with average linkage on the Spearman's correlations. The right corner, highlighted by the black square, shows the RCM (Ruminococcaceae Christensenellaceae Methanobrevibacter) cluster. The top row shows if the specific ASV was significant (FDR corrected $p < 0.05$) for each individual component of MetS based on the analysis from Figure 3. ASVs negatively associated with an outcome (either continuous, binary or both; indicated by different gradients) are indicated in green, while ASVs positively associated with an outcome are indicated in red. For HDL, the association is inverted, to make it more consistent with a healthier phenotype.

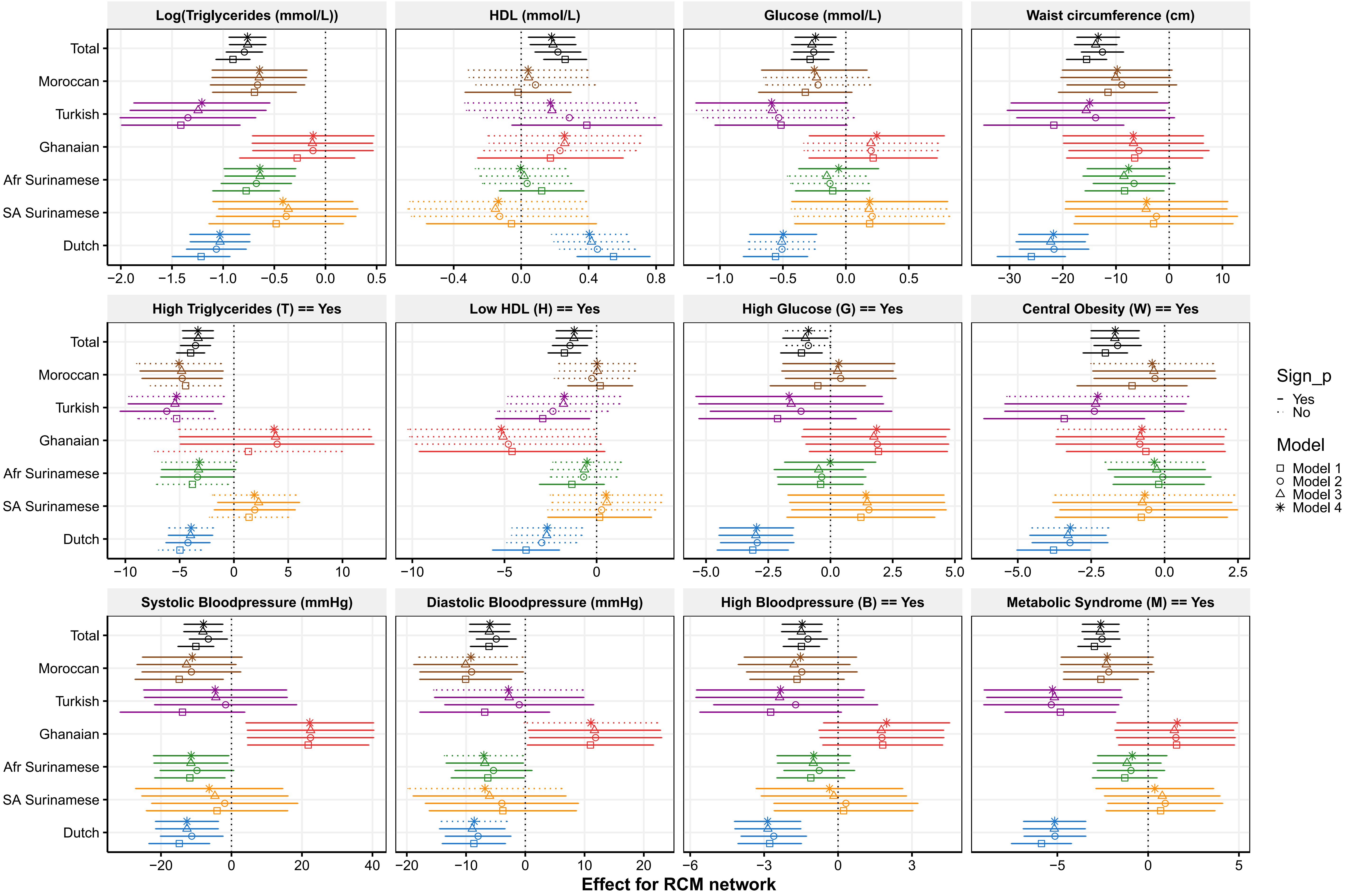

Fig. S7: Overview of the effects of the RCM cluster with 95% CI and p-values in the (logistic) regression models on MetS outcomes. For each model, each outcome measure was predicted with the arcsin squared-root transformed RCM cluster, sex, ethnicity (Dutch as reference), and sex:ethnicity (except High Triglycerides) and additional covariates. Those models represent the ethnic-independent effect (i.e. Total model). In addition, the effect per ethnicity is provided, which is derived from the model with an additional interaction term between ethnicity and the arcsin squared-root transformed RCM cluster abundance. Significance ($p < 0.05$; Sign_p) of this overall interaction term, assessed via LRT, is indicated by line type, as well as the significance of the overall effect of the RCM cluster in the Total model. Analyses were performed on the subcohort (n=3443) with microbiota data. For the binarized variables, logistic regression was performed and its effect is indicated by LogOdds ratio, while the others were analysed with a linear regression model and their effect is indicated by the coefficients in the model. Effects per ethnicity were calculated based on the coefficients and standard errors obtained from the int model output, including the coefficients and variance-covariance matrix. Covariates included in models: model 1: age; model 2: model 1 + PPI use + socioeconomic status; model 3: model 2 + lifestyle; model 4: model 3 + diet.

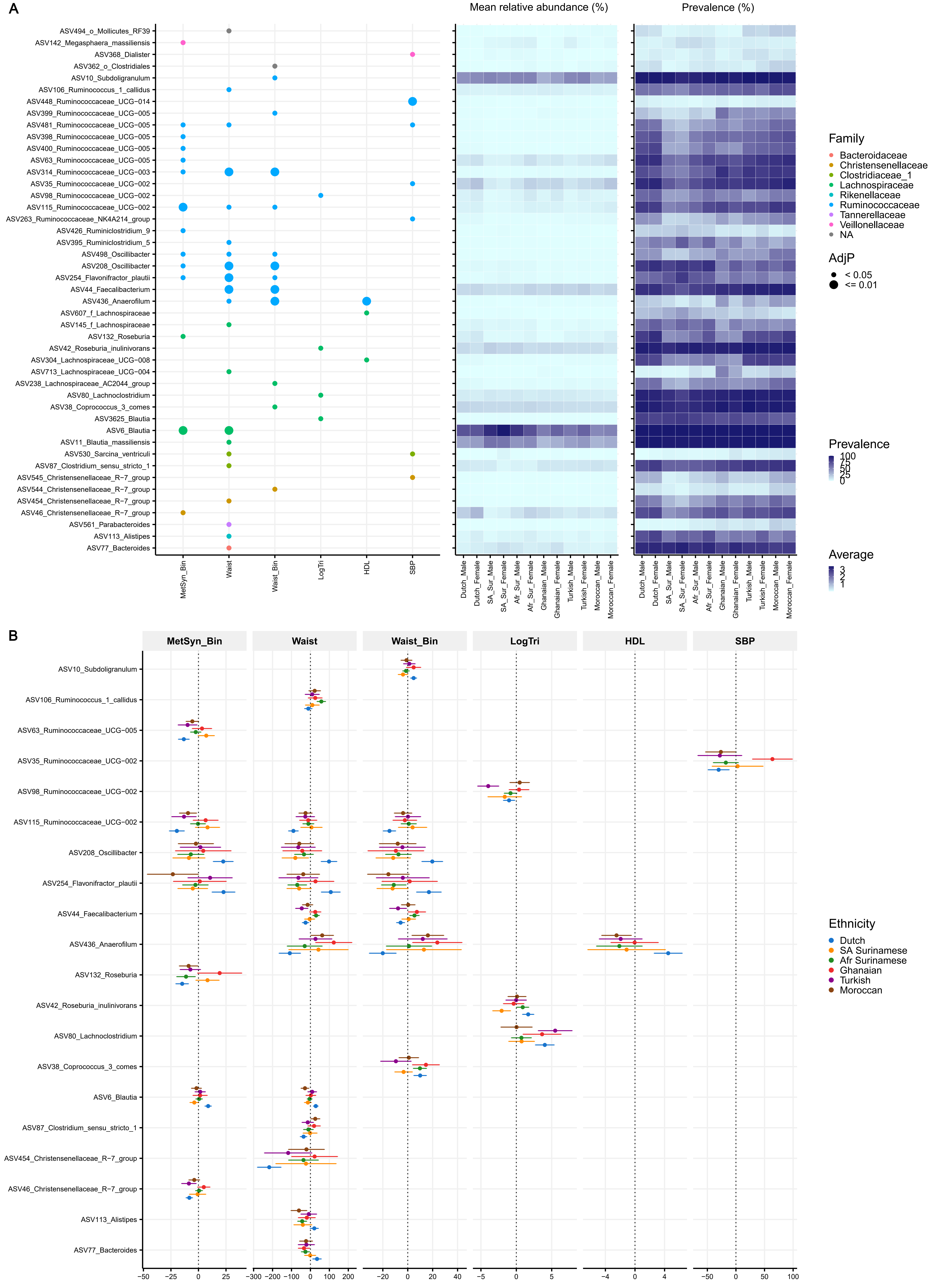

Fig. S8: Overview of ASVs with a statistically significant interaction term between ethnicity and an ASV per outcome, using (logistic) regression models. Models were run with the (arcsin-root transformed) ASV abundance:Ethnicity as independent variable, and adjusted for arcsin-root transformed ASV abundance, age, sex, ethnicity (Dutch as reference) and sex:ethnicity (except HighTri). Models and FDR correction was applied per outcome (either binarized or continuous). Interaction terms were tested with a likelihood ratio test. Analyses were performed on the subcohort (n=3443) with microbiota data. A) Overview of all ASVs with a significant interaction term (FDR corrected p < 0.05) indicated per outcome. B) Overview of the model effects (with 95% CI) per ethnicity for a subset of the significant ASVs. For binarized outcomes (indicated with 'Bin_'), the effect is indicated as a LogOdds ratio. For the continuous values, the effect is the coefficient from the model. Effects per ethnicity were calculated based on the coefficients and standard errors obtained from the int model output, including the coefficients and variance-covariance matrix.
