# Supplementary material for: Ethnic variations in metabolic syndrome components and their associations with the gut microbiota: the HELIUS study
Source: Genome Med. 2024 Mar 20;16:41. doi: 10.1186/s13073-024-01295-7 (PMC10953122; doi:10.1186/s13073-024-01295-7)
Supplement: Supplementary file 2 — Additional file 2: Table S1. Population characteristics for the subset of the cohort with gut microbiota data. Overview of population characteristics for the Dutch, South-Asian Surinamese (SA Surinamese), African Surinamese (Afr Surinamese), Ghanaian, Turkish and Moroccan, presented separately per sex. [file 13073_2024_1295_MOESM2_ESM.docx]

**Table S1:** Population characteristics for the subset of the cohort with gut microbiota data. Overview of population characteristics for the Dutch, South-Asian Surinamese (SA Surinamese), African Surinamese (Afr Surinamese), Ghanaian, Turkish and Moroccan, presented separately per sex.

|  | Dutch | SA Surinamese | Afr Surinamese | Ghanaian | Turkish | Moroccan |
| --- | --- | --- | --- | --- | --- | --- |
| **Males** |  |  |  |  |  |  |
| N | 560 | 139 | 341 | 130 | 169 | 293 |
| Age (in years) | 48.87 ± 12.81 | 46.01 ± 11.43 | 49.43 ± 10.86 | 48.80 ± 8.58 | 41.85 ± 10.73 | 43.82 ± 11.19 |
| Educational level Higher (%) | 60.5 | 27.3 | 20.2 | 6.2 | 19.5 | 16.4 |
| Intermediate (%) | 22.5 | 33.1 | 29.0 | 27.7 | 29.6 | 31.7 |
| Lower (%) | 13.8 | 29.5 | 46.0 | 46.9 | 29.6 | 26.6 |
| Elementary (%) | 2.7 | 10.1 | 4.1 | 16.9 | 19.5 | 23.5 |
| NA (%) | 0.5 | 0 | 0.6 | 2.3 | 1.8 | 1.7 |
| Occupational level Academic (%) | 19.8 | 9.4 | 2.3 | 0 | 3.6 | 3.4 |
| Higher (%) | 37.7 | 19.4 | 15.5 | 1.5 | 11.8 | 8.5 |
| Intermediate (%) | 21.4 | 25.9 | 24.6 | 11.5 | 23.7 | 22.2 |
| Lower (%) | 16.3 | 31.7 | 39.3 | 21.5 | 38.5 | 40.3 |
| Elementary (%) | 0.9 | 5.0 | 7.3 | 53.1 | 11.2 | 14.0 |
| NA (%) | 3.9 | 8.6 | 10.9 | 12.3 | 11.2 | 11.6 |
| Employment status Working (%) | 77.7 | 75.5 | 66.6 | 71.5 | 68.6 | 66.9 |
| Not in workforce (%) | 14.1 | 8.6 | 6.5 | 3.8 | 6.5 | 7.2 |
| Unemployed (%) | 5.2 | 8.6 | 19.1 | 20.8 | 14.8 | 15.7 |
| Unfit for work (%) | 2.9 | 6.5 | 5.9 | 2.3 | 7.7 | 9.6 |
| NA (%) | 0.2 | 0.7 | 2.1 | 1.5 | 2.4 | 0.7 |
| Smoking Yes (%) | 20.5 | 33.8 | 44.3 | 4.6 | 31.4 | 19.1 |
| Never (%) | 37.1 | 47.5 | 32.0 | 81.5 | 40.2 | 47.8 |
| Former (%) | 42.3 | 18.7 | 22.6 | 12.3 | 26.6 | 32.8 |
| NA (%) | 0 | 0 | 1.2 | 1.5 | 1.8 | 0.3 |
| Alcohol Yes (%) | 94.6 | 65.5 | 79.5 | 57.7 | 33.7 | 14.0 |
| No (%) | 5.4 | 34.5 | 19.9 | 40.0 | 65.1 | 85.3 |
| NA (%) | 0 | 0 | 0.6 | 2.3 | 1.2 | 0.7 |
| Physical activity Yes (%) | 73.4 | 69.1 | 70.7 | 60.8 | 47.3 | 56.0 |
| No (%) | 26.6 | 30.9 | 29.3 | 39.2 | 52.7 | 43.7 |
| NA (%) | 0 | 0 | 0 | 0 | 0 | 0.3 |
| SugarDrinks Yes (%) | 53.6 | 68.3 | 72.1 | 50.0 | 60.4 | 70.3 |
| No (%) | 46.3 | 30.2 | 26.4 | 48.5 | 36.7 | 28.3 |
| NA (%) | 0.2 | 1.4 | 1.5 | 1.5 | 3.0 | 1.4 |
| Fruit intake Yes (%) | 58.9 | 51.1 | 46.9 | 33.1 | 50.3 | 44.7 |
| No (%) | 40.9 | 48.9 | 52.2 | 65.4 | 48.5 | 53.9 |
| NA (%) | 0.2 | 0 | 0.9 | 1.5 | 1.2 | 1.4 |
| PPI use Yes (%) | 3.9 | 4.3 | 3.8 | 1.5 | 11.8 | 9.9 |
|  |  |  |  |  |  |  |
| MetSyn = Yes (%) | 26.1 | 32.4 | 19.1 | 13.1 | 36.7 | 30.7 |
| Central Obesity = Yes (%) | 45.2 | 53.2 | 33.7 | 40.0 | 64.5 | 57.7 |
| High Glucose = Yes (%) | 35.0 | 30.2 | 29.6 | 26.9 | 32.0 | 38.6 |
| High Blood pressure = Yes (%) | 50.2 | 46.0 | 56.0 | 69.2 | 47.3 | 42.7 |
| Low HDL = Yes (%) | 12.1 | 30.2 | 13.2 | 4.6 | 36.1 | 28.3 |
| High Triglycerides = Yes (%) | 16.7 | 24.5 | 10.0 | 3.8 | 26.6 | 14.7 |
|  |  |  |  |  |  |  |
| Waist Circumference (in cm) | 93.64 ± 11.15 | 91.56 ± 11.32 | 90.61 ± 11.88 | 90.38 ± 9.31 | 98.45 ± 11.11 | 96.15 ± 10.88 |
| Fasting Glucose (in mmol/L) | 5.41 ±0.45 | 5.40 ± 0.49 | 5.31 ± 0.46 | 5.25 ± 0.50 | 5.42 ± 0.46 | 5.46 ± 0.49 |
| SBP (in mmHg) | 129.5 ± 15.9 | 126.7 ± 17.3 | 131.9 ± 16.6 | 136.7 ± 17.8 | 124.9 ± 13.3 | 126.8 ± 15.0 |
| DBP (in mmHg) | 82.0 ± 9.9 | 82.1 ± 10.7 | 84.4 ± 9.7 | 87.3 ± 11.7 | 82.2 ± 9.6 | 79.9 ± 9.1 |
| HDL (in mmol/L) | 1.39 ± 0.37 | 1.18 ± 0.30 | 1.41 ± 0.41 | 1.51 ± 0.39 | 1.15 ± 0.30 | 1.19 ± 0.31 |
| Triglycerides (in mmol/L) | 0.99 [0.66 – 1.37] | 1.11 [0.73 – 1.67] | 0.79 [0.55 – 1.15] | 0.75 [0.52 – 1.01] | 1.14 [0.83 – 1.78] | 0.97 [0.7 – 1.4] |
|  |  |  |  |  |  |  |
| **Females** |  |  |  |  |  |  |
| N | 567 | 185 | 432 | 162 | 197 | 268 |
| Age (in years) | 48.96 ± 12.93 | 46.61 ± 12.06 | 47.57 ± 11.06 | 43.10 ± 9.45 | 41.03 ± 10.79 | 42.07 ± 10.69 |
| Educational level Higher (%) | 60.0 | 23.8 | 28.2 | 2.5 | 15.7 | 17.9 |
| Intermediate (%) | 21.0 | 28.1 | 40.3 | 16.0 | 36.5 | 31.7 |
| Lower (%) | 16.2 | 33.5 | 28.2 | 36.4 | 16.2 | 19.8 |
| Elementary (%) | 2.6 | 14.6 | 2.3 | 42.6 | 29.9 | 29.9 |
| NA (%) | 0.2 | 0 | 0.9 | 2.5 | 1.5 | 0.7 |
| Occupational level Academic (%) | 15.2 | 3.8 | 30.1 | 0 | 4.1 | 3.4 |
| Higher (%) | 38.4 | 14.6 | 21.1 | 1.9 | 6.6 | 13.8 |
| Intermediate (%) | 24.2 | 30.8 | 42.6 | 6.8 | 20.8 | 19.0 |
| Lower (%) | 14.8 | 31.9 | 22.7 | 15.4 | 26.4 | 17.2 |
| Elementary (%) | 2.8 | 9.2 | 3.0 | 66.7 | 15.2 | 14.6 |
| NA (%) | 4.6 | 9.7 | 7.6 | 9.3 | 26.9 | 32.1 |
| Employment status Working (%) | 72.0 | 70.8 | 68.1 | 58.6 | 47.7 | 40.7 |
| Not in workforce (%) | 19.2 | 14.6 | 11.1 | 3.1 | 27.9 | 32.1 |
| Unemployed (%) | 5.3 | 8.1 | 11.8 | 25.9 | 14.2 | 16.8 |
| Unfit for work (%) | 3.0 | 5.4 | 8.8 | 9.9 | 8.1 | 9.0 |
| NA (%) | 0.5 | 1.1 | 0.2 | 2.5 | 2.0 | 1.5 |
| Smoking Yes (%) | 17.8 | 14.6 | 20.4 | 1.2 | 27.4 | 3.4 |
| Never (%) | 39.5 | 74.1 | 60.9 | 93.2 | 59.9 | 91.4 |
| Former (%) | 42.5 | 10.8 | 18.3 | 4.3 | 11.7 | 5.2 |
| NA (%) | 0.2 | 0.5 | 0.5 | 1.2 | 1.0 | 0 |
| Alcohol Yes (%) | 88.4 | 47.6 | 69.2 | 37.7 | 15.7 | 3.0 |
| No (%) | 11.5 | 51.9 | 30.8 | 59.9 | 83.8 | 97.0 |
| NA (%) | 0.2 | 0.5 | 0 | 2.5 | 0.5 | 0 |
| Physical activity Yes (%) | 78.1 | 48.6 | 58.6 | 43.8 | 39.1 | 41.4 |
| No (%) | 21.9 | 51.4 | 41.4 | 56.2 | 60.9 | 58.6 |
| NA (%) | 0 | 0 | 0 | 0 | 0 | 0 |
| SugarDrinks Yes (%) | 38.1 | 55.1 | 50.9 | 52.5 | 51.3 | 63.8 |
| No (%) | 60.8 | 40.5 | 47.5 | 42.0 | 47.2 | 35.1 |
| NA (%) | 1.1 | 4.3 | 1.6 | 5.6 | 1.5 | 1.1 |
| Fruit intake Yes (%) | 71.3 | 56.8 | 55.8 | 38.3 | 58.4 | 56.0 |
| No (%) | 28.6 | 42.2 | 43.8 | 58.6 | 41.1 | 42.5 |
| NA (%) | 0.2 | 1.1 | 0.5 | 3.1 | 0.5 | 1.5 |
| PPI use Yes (%) | 6.0 | 4.3 | 6.3 | 3.1 | 11.2 | 11.6 |
|  |  |  |  |  |  |  |
| MetSyn = Yes (%) | 12.7 | 24.3 | 18.1 | 14.2 | 20.8 | 17.2 |
| Central Obesity = Yes (%) | 62.3 | 75.7 | 79.2 | 84.0 | 81.7 | 79.1 |
| High Glucose = Yes (%) | 15.9 | 25.9 | 15.0 | 16.7 | 14.2 | 19.8 |
| High Blood pressure = Yes (%) | 25.9 | 35.1 | 45.8 | 49.4 | 19.8 | 20.5 |
| Low HDL = Yes (%) | 12.9 | 33.0 | 20.8 | 10.5 | 34.0 | 36.2 |
| High Triglycerides = Yes (%) | 7.2 | 7.6 | 3.2 | 0 | 13.2 | 3.0 |
|  |  |  |  |  |  |  |
| Waist Circumference (in cm) | 85.95 ± 12.53 | 88.00 ± 11.71 | 91.32 ± 13.79 | 91.76 ± 12.14 | 91.09 ± 12.65 | 91.78 ± 13.58 |
| Fasting Glucose (in mmol/L) | 5.12 ± 0.47 | 5.22 ± 0.55 | 5.07 ± 0.47 | 5.08 ± 0.51 | 5.10 ± 0.44 | 5.13 ± 0.50 |
| SBP (in mmHg) | 120.7 ± 16.0 | 123.4 ± 18.0 | 126.9 ± 17.1 | 131.0 ± 18.8 | 117.1 ± 13.7 | 117.4 ± 15.6 |
| DBP (in mmHg) | 75.0 ± 9.2 | 77.1 ± 9.9 | 80.0 ± 10.7 | 81.6 ± 10.8 | 74.5 ± 8.6 | 73.0 ± 8.5 |
| HDL (in mmol/L) | 1.77 ± 0.44 | 1.49 ± 0.36 | 1.64 ±0.43 | 1.75 ± 0.42 | 1.48 ± 0.36 | 1.48 ±0.39 |
| Triglycerides (in mmol/L) | 0.74 [0.53 – 1.08] | 0.87 [0.61 – 1.29] | 0.65 [0.50 – 0.89] | 0.56 [0.39 – 0.73] | 0.83 [0.55 – 1.20] | 0.75 [0.50 – 1.00] |
